# A Mendelian randomization study of the effect of body mass index on 52 causes of death among 125 000 Mexican adults with admixed ancestry

**DOI:** 10.1093/ije/dyaf110

**Published:** 2025-07-11

**Authors:** Louisa Gnatiuc Friedrichs, Pablo Kuri-Morales, Eirini Trichia, Natalie Staplin, Jason Torres, Jesus Alegre-Díaz, Paulina Baca, Adrián Garcilazo-Ávila, Carlos González-Carballo, Raul Ramirez-Reyes, Fernando Rivas, Diego Aguilar-Ramirez, Fiona Bragg, Robert Clarke, William G Herrington, Michael Hill, Tianshu Liu, Alejandra Vergara-Lope, Rachel Wade, Rory Collins, Richard Peto, Jaime Berumen, Roberto Tapia-Conyer, Jonathan R Emberson

**Affiliations:** Clinical Trial Service Unit and Epidemiological Studies Unit, Nuffield Department of Population Health, University of Oxford, Oxford, United Kingdom; School of Medicine, National Autonomous University of Mexico, Mexico City, Mexico; Instituto Tecnológico y de Estudios Superiores de Monterrey, Monterrey, Mexico; Clinical Trial Service Unit and Epidemiological Studies Unit, Nuffield Department of Population Health, University of Oxford, Oxford, United Kingdom; Clinical Trial Service Unit and Epidemiological Studies Unit, Nuffield Department of Population Health, University of Oxford, Oxford, United Kingdom; Clinical Trial Service Unit and Epidemiological Studies Unit, Nuffield Department of Population Health, University of Oxford, Oxford, United Kingdom; Experimental Research Unit from the Faculty of Medicine, National Autonomous University of Mexico, Mexico City, Mexico; Experimental Research Unit from the Faculty of Medicine, National Autonomous University of Mexico, Mexico City, Mexico; Experimental Research Unit from the Faculty of Medicine, National Autonomous University of Mexico, Mexico City, Mexico; Experimental Research Unit from the Faculty of Medicine, National Autonomous University of Mexico, Mexico City, Mexico; Experimental Research Unit from the Faculty of Medicine, National Autonomous University of Mexico, Mexico City, Mexico; Experimental Research Unit from the Faculty of Medicine, National Autonomous University of Mexico, Mexico City, Mexico; Clinical Trial Service Unit and Epidemiological Studies Unit, Nuffield Department of Population Health, University of Oxford, Oxford, United Kingdom; Clinical Trial Service Unit and Epidemiological Studies Unit, Nuffield Department of Population Health, University of Oxford, Oxford, United Kingdom; Health Data Research UK Oxford, University of Oxford, Oxford, United Kingdom; Clinical Trial Service Unit and Epidemiological Studies Unit, Nuffield Department of Population Health, University of Oxford, Oxford, United Kingdom; Clinical Trial Service Unit and Epidemiological Studies Unit, Nuffield Department of Population Health, University of Oxford, Oxford, United Kingdom; Clinical Trial Service Unit and Epidemiological Studies Unit, Nuffield Department of Population Health, University of Oxford, Oxford, United Kingdom; Clinical Trial Service Unit and Epidemiological Studies Unit, Nuffield Department of Population Health, University of Oxford, Oxford, United Kingdom; Clinical Trial Service Unit and Epidemiological Studies Unit, Nuffield Department of Population Health, University of Oxford, Oxford, United Kingdom; Clinical Trial Service Unit and Epidemiological Studies Unit, Nuffield Department of Population Health, University of Oxford, Oxford, United Kingdom; Clinical Trial Service Unit and Epidemiological Studies Unit, Nuffield Department of Population Health, University of Oxford, Oxford, United Kingdom; Clinical Trial Service Unit and Epidemiological Studies Unit, Nuffield Department of Population Health, University of Oxford, Oxford, United Kingdom; Experimental Research Unit from the Faculty of Medicine, National Autonomous University of Mexico, Mexico City, Mexico; School of Medicine, National Autonomous University of Mexico, Mexico City, Mexico; Clinical Trial Service Unit and Epidemiological Studies Unit, Nuffield Department of Population Health, University of Oxford, Oxford, United Kingdom

**Keywords:** Mendelian randomization, body mass index, mortality, prospective study, Mexico

## Abstract

**Background:**

Persistent hyperglycaemia in diabetes can cause weight loss, distorting the association of adiposity with mortality. We estimated the lifelong associations of genetically predicted body mass index (BMI) with 52 causes of death among 125 003 Mexican adults, in whom persistent hyperglycaemia in diabetes was common.

**Methods:**

A trans-ancestry genetic instrument for BMI (from 724 BMI-associated single-nucleotide polymorphisms) estimated the causal relevance of BMI to mortality before age 75 years, stratified by sex and adjusted for age and underlying ancestry structure, using a one-sample Mendelian randomization (MR) approach. Two-sample MR and other sensitivity analyses were also performed.

**Results:**

The genetic instrument explained 3% of the BMI variation and predicted BMI similarly in men and women. Each 5-kg/m^2^ higher genetically predicted BMI was associated with nearly a doubling in the risk of all-cause mortality at ages 35–74 years [13 066 deaths; hazard ratio (HR) 1.80, 95% confidence interval (CI) 1.63–2.00]. Hazard ratios were greater for vascular-metabolic (*n *= 7111; HR 2.15, 95% CI 1.87–2.48) than for non-vascular-metabolic causes (*n *= 5955; HR 1.47, 95% CI 1.27–1.71) and particularly strong for renal (*n *= 2034; HR 3.59, 95% CI 2.76–4.67), acute diabetic crises (*n *= 557; HR 2.70, 95% CI 1.64–4.44), and infective deaths (*n *= 811; HR 2.61, 95% CI 1.73–3.92). For all-cause mortality, HRs were somewhat greater at younger ages compared with older ages, and slightly larger in those with a higher proportion of Indigenous American ancestry. The strength of the association with mortality was reduced by more than half after simple adjustment for genetic predisposition to diabetes. Sensitivity analyses supported the main conclusions.

**Conclusion:**

In this Mexican population, genetically predicted lifelong BMI was strongly related to mortality and mediated substantially through diabetes.

Key Messages

- Obesity is a global public health concern, but there is little evidence on the causal effects of body mass index (BMI) on mortality in Mexico.
- Using a Mendelian randomization approach applied to a large Mexican prospective cohort study, we found that 5-kg/m^2^ higher genetically predicted BMI was associated with nearly a doubling in all-cause mortality rates at ages 35–74 years, with particularly strong effects for vascular-metabolic causes as well as infection. At least half of the effect of BMI on mortality was explained by its effect on diabetes.
- These estimated causal effects of BMI on mortality risk are larger than have been reported previously, reinforcing the need in Mexico for strategies to reduce population-wide levels of adiposity and optimize the use of appropriate risk-reducing therapies.

## Introduction

Adiposity and diabetes are major causes of premature death and disability worldwide, and both are particularly common in Mexico [1, 2]. Excess adiposity causes diabetes, but persistent hyperglycaemia in diabetes can itself cause pathological weight loss, distorting the observed associations of adiposity with prevalent diabetes and with cause-specific mortality [3]. Previous analyses of the Mexico City Prospective Study (MCPS)—a cohort of 150 000 Mexican adults recruited 20 years ago—found that hyperglycaemia in diabetes was common and associated with a quadrupling in all-cause mortality at ages 35–74 years [4]. Subsequent analyses found that, among individuals without diabetes, the association between body mass index (BMI) and mortality was J-shaped (comparable to that observed in other populations [5]), with each 5-kg/m^2^ higher BMI above 25 kg/m^2^ associated with a 30% higher all-cause mortality [3]. However, among those with diabetes, BMI was inversely associated with mortality, likely due to reverse causality. Participants with diabetes were therefore excluded from the observational analyses of adiposity and mortality, but this exclusion may have resulted in underestimation of the full effect of adiposity on mortality by removing, at least in part, the impact of adiposity on mortality that is mediated through diabetes.

Mendelian randomization (MR) approaches can mitigate biases due to reverse causality and confounding that are inherent in observational studies and seek to estimate the effects of lifelong differences in exposures on disease risks [6]. Previous MR studies conducted mostly in populations of European ancestry support the causal relevance of adiposity to cardiometabolic diseases and all-cause mortality [7–9] and have identified individual diseases most strongly associated with adiposity [10, 11]. However, evidence from non-European populations with admixed ancestry remains scarce. To address this gap, we used MR approaches to estimate the causal relevance of lifelong differences in BMI for cause-specific mortality among 125 000 Mexican adults with admixed ancestry previously enrolled into the MCPS [12].

## Methods

The MCPS study design and methods have been described previously [12, 13]. A detailed description of the exclusions, genetic instruments for BMI and diabetes, mortality follow-up procedures and outcomes, and statistical analyses used in the current paper is provided in the Methods section of the Supplementary Material. A brief summary of the statistical methods is given below.

An additive genetic model of inheritance was assumed throughout. The one-sample Wald ratio method was used to assess the causal relevance of BMI to cause-specific mortality [14], in which Cox regression was used to estimate the association between a trans-ancestry BMI genetic score (BMI-GS) [15] and mortality, and linear regression was used to estimate the association between the BMI-GS and BMI. Analyses were performed separately for men and women, with the inverse-variance-weighted average of the resulting log hazard ratios (HRs) used when combining men and women. The assumptions of MR are that: (i) the instrument is associated with the exposure; (ii) the instrument is not associated with genetic confounders; and (iii) the instrument does not affect the outcome except through the exposure. The first assumption was assessed by using analysis of variance to estimate the percentage of variance in the observed BMI explained by the BMI-GS and, also, by plotting the mean BMI across fifths of the BMI-GS. The second assumption was addressed by adjusting for genetic principal components. To examine the possibility of violations against the third assumption, we tabulated the mean levels of potential confounders across fifths of the BMI-GS and performed additional ‘two-sample’ MR approaches (including weighted median and MR–Egger) [16].

To explore the extent to which the effect of genetically predicted BMI on mortality could be mediated by genetic diabetes liability, the direct causal effects of BMI were estimated in a mediation MR framework in which a trans-ancestry diabetes GS was constructed (from a set of genetic variants that did not overlap with the variants in the BMI-GS). A two-stage least-squares mediation MR approach was then used in which genetically predicted BMI and genetically predicted diabetes liability were jointly entered into a Cox model to estimate the causal effect of BMI on mortality not mediated by diabetes [17]. The proportion mediated was estimated by subtracting the log HR of the direct effect from the log HR of the total effect and then dividing by the log HR of the total effect. Bootstrap resampling (with 1000 resamples) was then used to provide a bias-corrected 95% confidence interval (CI) for this proportion.

Sensitivity analyses included: restriction to participants unrelated to the third family degree; use of a relaxed ‘clumping’ threshold when constructing the BMI-GS; excluding those with pre-existing diabetes or other chronic disease when estimating the association between the BMI-GS and BMI; alternative use of a BMI-GS derived from a large European ancestry population published by the GIANT consortium; analyses of mortality at younger versus older ages, by residential district, by proportion of Indigenous American ancestry, and by levels of physical activity and smoking status; and use of non-linear MR (both residual and doubly ranked methods [18, 19]) to explore the log-linearity assumption between genetically predicted BMI and mortality risk. Non-linear MR analyses with the ‘negative controls’ of age and sex were also performed to explore the exclusion restriction assumption of MR.

Analyses were conducted by using STATA (version 17), SAS (version 9.4), and R (version 3.3.0).

## Results

### Study population and baseline characteristics

Of 159 755 participants recruited, 25 845 (16%) were excluded from all analyses. These comprised 21 244 (13%) without quality-control (QC) acceptable genetic data (see Methods section of the Supplementary Material), a further 729 (0.4%) aged ≥90 years at recruitment, a further 1617 (1%) with missing or implausible height or weight measurements, and a further 2255 (1%) with uncertain mortality linkage. Of the remaining 133 910 participants, 125 003 were aged 35–74 years at recruitment [mean (SD) age 50 (11) years, 68% women] and 8907 were aged 75–89 years at recruitment. Of the 125 003 aged 35–74 years, the average ancestry proportions were 67% Indigenous American, 28% European, 3% African, and 1% East Asian, and 71 513 (57%) were unrelated to the third family degree. The baseline characteristics of the 125 003 participants aged 35–74 years at recruitment are shown in Table 1.

**Table 1.** Baseline characteristics of men and women aged 35–74 years

	Men	Women	All
	**(*n* = 40** **311)**	**(*n* = 84** **692)**	**(*n* = 125** **003)**
Age (years)	50 (11)	50 (11)	50 (11)
Percentage of Indigenous American ancestry	67 (54–81)	67 (54–81)	67 (54–81)
Socioeconomic status and lifestyle behaviours			
Resident of Coyoacán	16 870 (42)	31 312 (37)	48 182 (39)
University/high-school educated	9970 (25)	10 055 (12)	20 025 (16)
Current smoker	20 532 (51)	20 163 (24)	40 695 (33)
Current drinker	31 251 (78)	53 259 (63)	84 510 (68)
Any regular leisure-time physical activity	12 198 (30)	15 843 (19)	28 041 (22)
Physical measurements			
Height (cm)	165 (6.9)	152 (6.3)	156 (8.9)
Weight (kg)	76 (12.8)	68 (12.7)	71 (13.3)
BMI (kg/m^2^)	28.0 (4.2)	29.7 (5.2)	29.1 (5.0)
Waist–hip ratio	0.95 (0.066)	0.88 (0.069)	0.90 (0.077)
Systolic blood pressure (mmHg)	128 (15)	126 (17)	127 (16)
Diastolic blood pressure (mmHg)	84 (10)	82 (10)	83 (10)
Diabetes			
Previously diagnosed diabetes^a^	5377 (13)	11 047 (13)	16 424 (13)
Undiagnosed diabetes^b^	2177 (5)	4314 (5)	6491 (5)
Subtotal: any diabetes	7554 (19)	15 361 (18)	22 915 (18)
HbA_1c_ in those with diabetes (%)	8.9 (6.8–10.7)	9.0 (6.9–10.8)	8.9 (6.9–10.8)
HbA_1c_ in those without diabetes (%)	5.5 (5.3–5.7)	5.5 (5.2–5.7)	5.5 (5.2–5.7)
Other diseases and long-term medication			
Other chronic disease^c^	1769 (4)	3640 (4)	5409 (4)
Any antihypertensive	4253 (11)	13 826 (16)	18 079 (14)
Any antithrombotic	1070 (3)	2375 (3)	3445 (3)
Any lipid-lowering	254 (<1)	432 (<1)	686 (<1)

Data are *n* (%), mean (SD), or median (interquartile range). HbA_1c_, glycosylated haemoglobin.

a Self-reported diagnosis or receiving a diabetes medication.

b Not previously diagnosed but HbA_1c_ ≥6.5%.

c Cardiovascular disease, stroke, chronic kidney disease, liver cirrhosis, cancer, or chronic obstructive pulmonary disease.

### Genetic instrument for BMI

The BMI-GS created from the trans-ancestry meta-analysis comprised 724 single-nucleotide polymorphisms (SNPs). The per-allele effects of these SNPs on BMI in MCPS were generally consistent with the estimates provided by the trans-ancestry meta-analysis (Supplementary Figure S1). The BMI-GS explained 3% of the variance in measured BMI in both men (F-statistic 1178) and women (F-statistic 2866). A 1-SD higher BMI-GS predicted a 0.70-kg/m^2^ higher BMI in men and a 0.94-kg/m^2^ higher BMI in women (Supplementary Figure S2). In both men and women, these slopes were slightly shallower in older participants and in those with the highest proportion of Indigenous American ancestry. Across fifths of the sex-specific BMI-GS distributions, BMI increased from 27.1 ± 4.0 to 29.0 ± 4.5 kg/m^2^ in men and from 28.4 ± 4.8 to 31.0 ± 5.4 kg/m^2^ in women (Supplementary Table S1). In both men and women, the mean systolic blood pressure increased by an average of 2 mmHg across fifths of the BMI-GS, while the prevalence of diabetes increased by 5 percentage points. Other characteristics did not vary much by the BMI-GS.

### Genetically predicted BMI and all-cause, vascular-metabolic, and non-vascular-metabolic mortality

**Figure 1.**
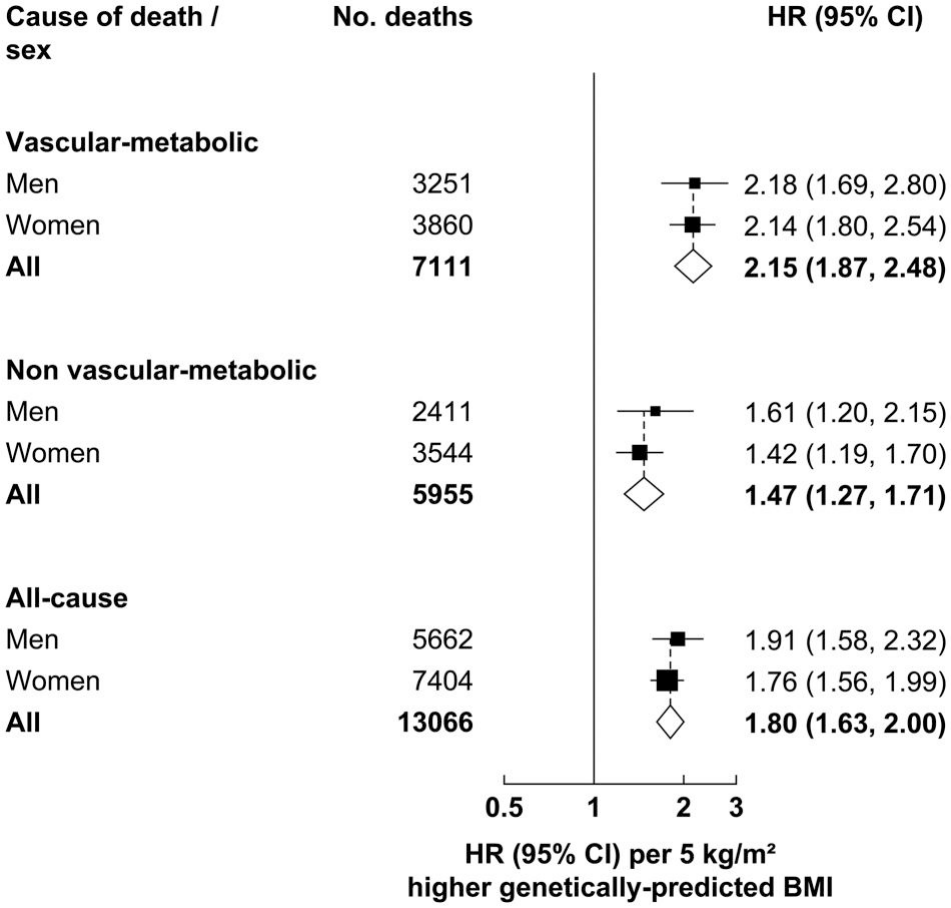
Association of genetically predicted BMI with all-cause, vascular-metabolic, and non-vascular-metabolic mortality at ages 35–74 years, overall and by sex. HR estimates for men and women (and their 95% CIs) calculated using the one-sample ratio method and shown per 5-kg/m^2^ higher genetically predicted BMI. The area of each square is proportional to the amount of statistical information (i.e. inversely proportional to the variance of the log HR). The overall estimates (shown as diamonds) are the inverse-variance-weighted averages of the sex-specific HRs.

During a median of 20 years of follow-up, a total of 13 066 deaths occurred at ages 35–74 years, including 7111 vascular-metabolic deaths (3451 vascular, 2034 renal, 1069 hepatobiliary, and 557 acute diabetic crises) and 5955 non-vascular-metabolic deaths (2107 cancer, 2050 respiratory, 811 infective, and 987 external, other, or ill-defined; see Supplementary Table S2 for the International Classification of Diseases Tenth Revision codes used). An increase of 5 kg/m^2^ in genetically predicted BMI was associated with nearly a doubling in all-cause mortality (HR 1.80, 95% CI 1.63–2.00) (Figure 1). For the composite of all vascular-metabolic deaths, the HR per 5-kg/m^2^ increase in genetically predicted BMI was 2.15 (95% CI 1.87–2.48) while, for the composite of all non-vascular-metabolic deaths, it was 1.47 (95% CI 1.27–1.71). Estimates were similar in men and women.

Sensitivity analyses in which a less stringent clumping threshold was used, or in which those with diabetes or other chronic disease were excluded from the estimated association of the BMI-GS with BMI, or that were based on a BMI-GS derived from the GIANT consortium genome wide association study (GWAS) gave broadly similar estimates (Supplementary Figures S3–S5, though the HRs when using the GIANT consortium GWAS were a little higher). Two-sample MR analyses also gave broadly similar results, albeit with slightly weaker HR estimates (Supplementary Table S3 and Supplementary Figure S6). Use of the residual and doubly ranked non-linear methods supported the plausibility of the associations between the genetically predicted BMI and all-cause, vascular-metabolic, and non-vascular-metabolic being continuous and log-linear (Supplementary Figure S7); however, the same methods applied to age and sex revealed non-zero associations (Supplementary Figure S8), illustrating the importance of fully controlling for the effects of age and sex in the main analyses.

### Genetically predicted BMI and specific causes of death

**Figure 2.**
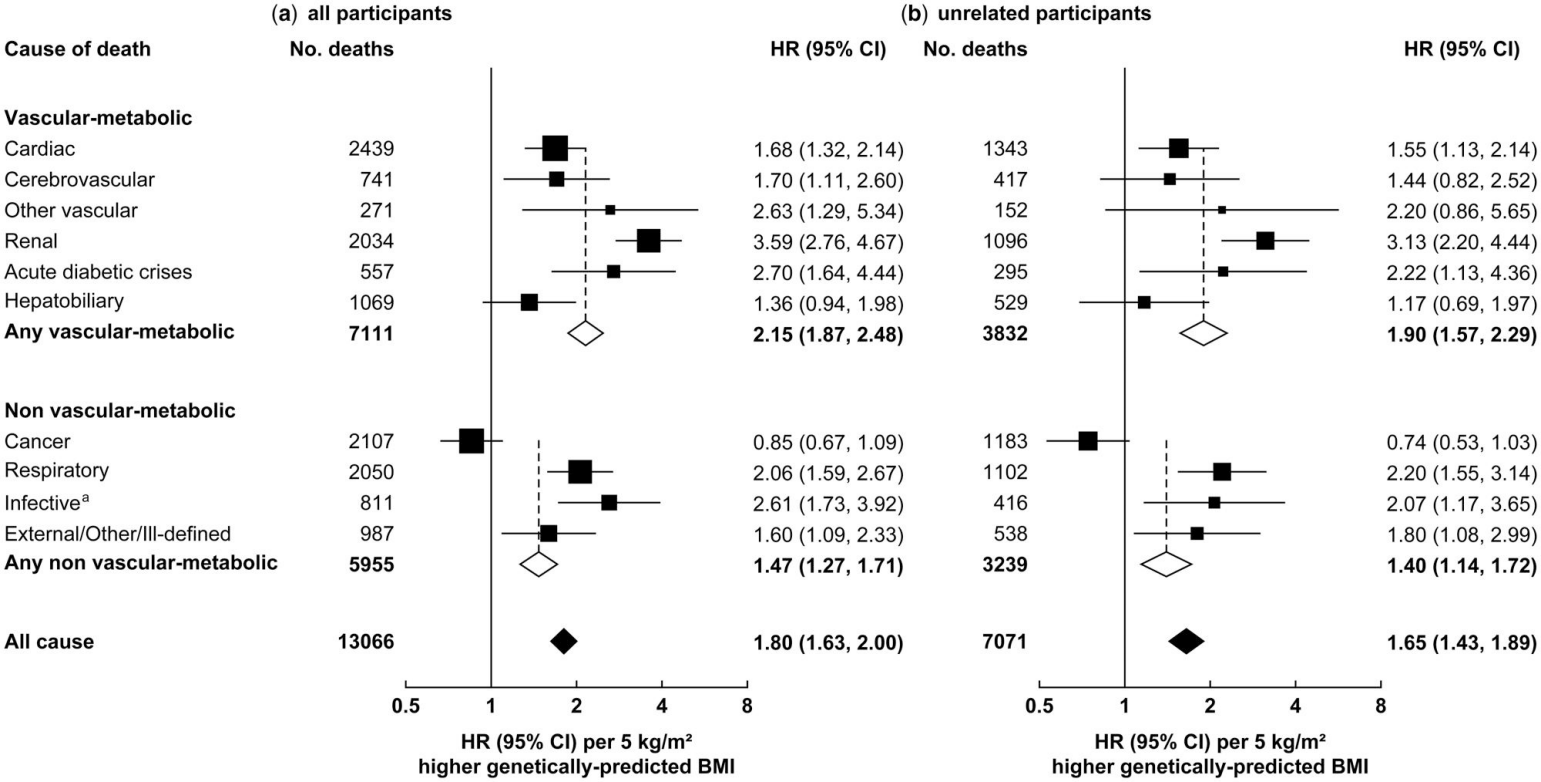
Association of genetically predicted BMI with vascular-metabolic and non-vascular-metabolic causes of death at ages 35–74 years, among (a) all participants and (b) participants unrelated to the third degree. ^a^Excluding infections included in other shown categories. Analyses and conventions as for Figure 1.

**Figure 3.**
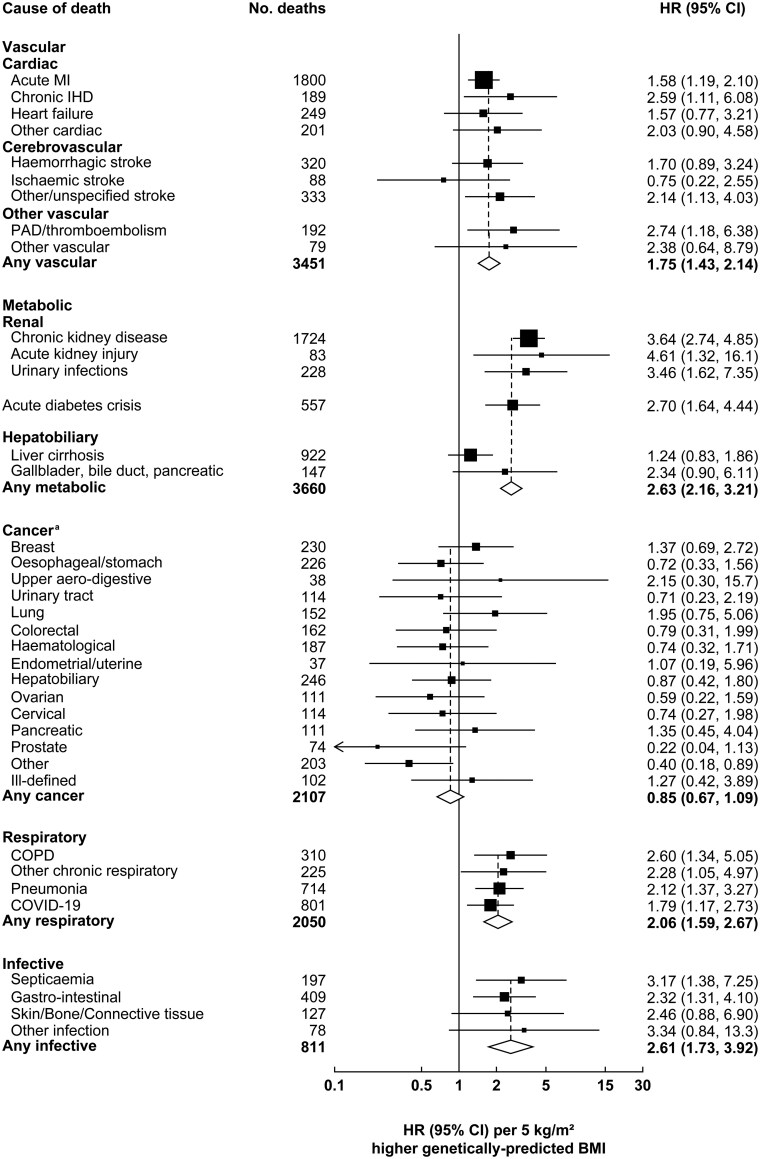
Association of genetically predicted BMI with subtypes of vascular-metabolic and non-vascular-metabolic causes of death at ages 35–74 years. ^a^Analyses of breast, endometrial/uterine, ovarian, and cervical cancer were performed only in women, while analyses of prostate cancer were performed only in men. One man who died from breast cancer is included in the ‘other’ cancer category. Analyses and conventions as for Figure 1. COPD, chronic obstructive pulmonary disease; COVID-19, coronavirus disease 2019; IHD, ischaemic heart disease; MI, myocardial infarction; PAD, peripheral arterial disease.

Of the individual causes of death studied, genetically predicted BMI was particularly strongly related to renal death (HR per 5-kg/m^2^ higher genetically predicted BMI 3.59, 95% CI 2.76–4.67) and acute diabetic crises (HR 2.70, 95% CI 1.64–4.44) (Figure 2). The HR was 1.68 (95% CI 1.32–2.14) for cardiac death and 1.70 (95% CI 1.11–2.60) for cerebrovascular death. Three-quarters of cardiac deaths were due to acute myocardial infarction (HR 1.58, 95% CI 1.19–2.10), with heart failure the second-most common cardiac cause (HR 1.57, 95% CI 0.77–3.21) (Figure 3). For all vascular deaths combined, the HR was 1.75 (95% CI 1.43–2.14). Of the non-vascular-metabolic causes of death, genetically predicted BMI was particularly strongly related to respiratory death (HR 2.06, 95% CI 1.59–2.67) and infective deaths (HR 2.61, 95% CI 1.73–3.92), but it was not positively related to cancer (HR 0.85, 95% CI 0.67–1.09), overall or for specific sites. When analyses were rerun in the subset of participants unrelated to the third degree, associations with particular causes of death were marginally weaker, albeit with wider CIs (Figure 2).

### Impact of other characteristics

The relevance of genetically predicted BMI to all-cause mortality was somewhat greater at younger ages compared with older ages (HR 2.43, 95% CI 2.01–2.93 at ages 35–59 years versus HR 1.43, 95% CI 1.17–1.74 at ages 70–74 years) and marginally greater in those with a higher proportion of inherited Indigenous American ancestry (HR 2.01, 95% CI 1.69–2.39 in those in the top third of Indigenous American ancestry versus HR 1.57, 95% CI 1.30–1.89 in those in the bottom third) [Supplementary Figure S9a: use of sex- and subgroup-specific estimates of the BMI-GS-to-BMI association gave nearly identical results (Supplementary Figure S9b)]. The relevance of genetically predicted BMI to all-cause mortality was similar irrespective of residential district, smoking, and physical activity status. Among all 133 910 participants aged <90 years at recruitment, the all-cause mortality HR associated with 5-kg/m^2^ higher genetically predicted BMI at ages 75–89 years was 1.40 (95% CI 1.22–1.60) (Supplementary Figure S10).

### Mediation through genetic diabetes liability

**Figure 4.**
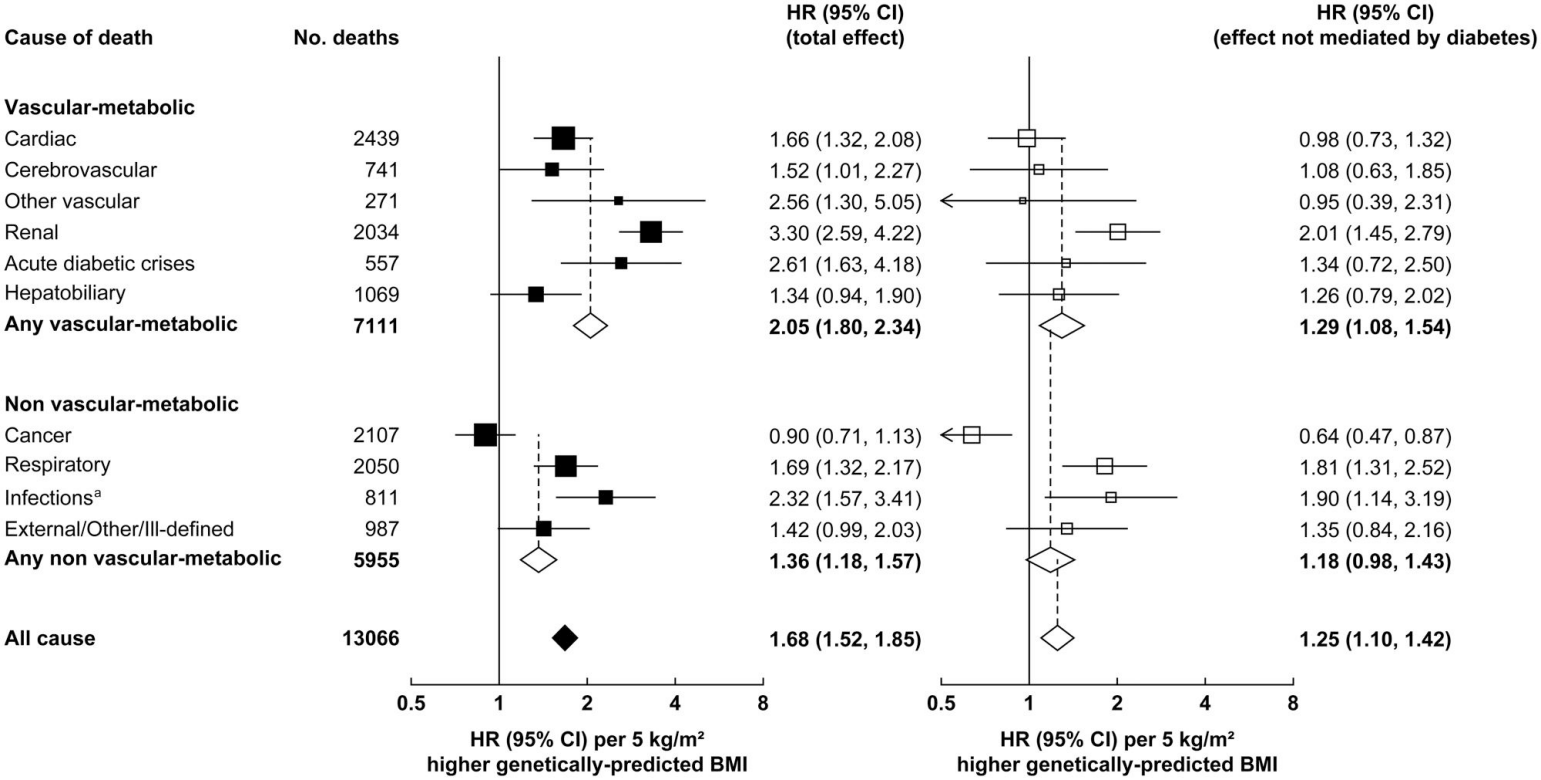
Two-stage least-squares estimates of the association of genetically predicted BMI with vascular-metabolic and non-vascular-metabolic causes of deaths at ages 35–74 years, before and after adjustment for genetic liability to diabetes. ^a^Excluding infections included in other shown categories. Analyses use the two-stage least-squares method (rather than the Wald ratio method) to estimate: (a) the total effect of genetically predicted BMI on mortality; and (b) the direct effect that is not mediated by diabetes.

As noted above, the prevalence of diabetes increased strongly across fifths of the BMI-GS. Figure 4 shows estimates of genetically predicted BMI with cause-specific mortality by using the two-stage least-squares (2SLS) method—rather than the Wald ratio method—before and after adjustment for genetic diabetes liability. As expected, the HRs from the 2SLS-based analyses were similar (albeit marginally weaker) to those from the Wald ratio method. Adjustment for genetic diabetes liability reduced the magnitude of the all-cause mortality log HR associated with the genetically predicted BMI by more than half [from ln(1.68) to ln(1.25) for all-cause mortality—a 56% reduction in magnitude (95% CI 35% to 94%)]. For vascular-metabolic mortality, the reduction in the log HR ratio on the adjustment for genetic diabetes liability was about two-thirds while, for the composite of all non-vascular-metabolic mortality, it was just under half.

## Discussion

In this large admixed Mexican population with high levels of obesity, we found strong evidence supporting a causal relationship between BMI and death from vascular, renal, diabetic, respiratory, and infective causes. Each 5-kg/m^2^ higher genetically predicted lifelong BMI conferred more than a doubling in the risk of vascular-metabolic mortality, including more than a trebling in risk of renal mortality. For all-cause mortality, there was a clear trend towards larger proportional effects at younger ages and a suggestive trend of larger proportional effects in those with a higher proportion of Indigenous American ancestry. Effects were similar in men and women, however.

The present MR analyses aim to estimate the lifelong causal effect of BMI on mortality risk. By contrast, previous observational analyses from the same cohort estimated the relationship between measured BMI during middle age and cause-specific mortality [3]. In those analyses, individuals with diabetes at recruitment (as well as deaths during the first 5 years of follow-up) were excluded in order to reduce the reverse-causality bias caused by persistent hyperglycaemia in diabetes resulting in weight loss prior to BMI measurement at recruitment. After such exclusions, a J-shaped association between BMI and mortality was observed in which each 5-kg/m^2^ higher BMI above 25 kg/m^2^ was associated with 30% higher risk of all-cause mortality and 40% higher risk of vascular-metabolic mortality (which is consistent with that seen in other populations worldwide) [3, 5]. However, although the exclusion of individuals with diabetes in our previous analyses was deemed necessary to limit reverse causality, it follows that the lifelong effects of BMI on mortality that are mediated ‘through’ diabetes would not have been fully captured in that analysis. This may well explain the larger relative risks of all-cause mortality associated with higher genetically predicted BMI in the current report. Indeed, the mediation analyses in the current report for the effect of BMI on mortality ‘not’ mediated through diabetes are consistent with our previous observational analyses [3]. Taken together, the results suggest that BMI may be an even more important cause of death in Mexico than previously estimated.

The magnitude of the associations reported in the current analysis of this Mexican population is somewhat greater than reported in previous MR studies conducted in European ancestry populations. For example, in a meta-analysis of 14 prospective studies and randomized trials, and four consortia enriched for coronary heart disease (CARDIoGRAMplusC4D), stroke (METASTROKE), diabetes (DIAGRAM), and lipids (GLGC) [11], each 4.6-kg/m^2^ higher BMI was associated with a 36% increase in the odds of coronary heart disease and 98% increase in the odds of diabetes, but only a 9% increase in the odds of stroke. Likewise, analyses of 14 cardiovascular diseases in 370 000 UK Biobank participants found that each 5-kg/m^2^ higher genetically predicted BMI was associated with about a 40% increase in the odds of coronary artery disease and 16% increase in the odds of stroke, with the strongest vascular associations observed for deep vein thrombosis (61% increase), heart failure (76% increase), and aortic stenosis (84% increase) [10]. A subsequent meta-analysis of MR studies plus de novo analyses of the FinnGen cohort confirmed the associations previously reported for diabetes and other cardiovascular diseases, but also demonstrated associations with several other diseases [20]. For example, in that study, each 1-SD higher genetically predicted BMI was associated with a 64% increase in the odds of gallstone disease, 81% increase in the odds of non-alcoholic fatty liver disease, and 65% increase in the odds of chronic obstructive pulmonary disease, as well as an increase in the odds of several cancers [20]. Positive associations between genetically predicted BMI and disease risk have also been reported for infections at multiple sites, at least at BMI levels of >20 kg/m^2^ [21].

Established mechanisms through which higher BMI increases cardiovascular and other diseases include insulin resistance, blood pressure, dyslipidaemia, and inflammation [22]. Mechanisms for the increased risk of infections associated with obesity include insulin resistance, but may also include immune function and adaptive responses [23], while pathways for the increased risk of chronic lung disease are less well understood. Causal associations between higher adiposity and adverse effects on glycaemia, blood pressure, and lipids have been confirmed by MR studies [11, 24], while randomized trials of weight loss have demonstrated that these adverse effects can be reversed [25–27]. Clinical guidelines, both in Mexico and elsewhere, have prioritized the avoidance of excess weight (among other risk factors) for preventing premature cardiovascular events [28, 29]. The findings of the present study provide strong support for recommendations prioritizing adiposity (and diabetes) control for the prevention of cardiometabolic diseases in Mexico.

The chief strengths of the present study include the large size, prolonged follow-up, and the fact that it was conducted in a previously understudied ancestry group with high levels of obesity and diabetes. The availability of genetic data in all participants allowed the use of a ‘one-sample’ MR approach. One advantage of this approach over alternative ‘two-sample approaches’ is that the instrument-to-BMI and instrument-to-mortality associations are estimated in the ‘same’ underlying population. The use of a single allele score in preference to each genetic variant acting as a separate instrumental variable (as done in the sensitivity analyses) also helped to reduce any weak-instrument bias. The primary instrument was derived from a trans-ancestry GWAS meta-analysis and, consequently, there was no overlap between MCPS participants and participants in the studies used to identify the SNPs used in the instrument or determine their weights. However, it did not include much Hispanic population representation, which may explain why the instrument explained only a fairly small proportion of BMI variance. Nonetheless, the strength of association between the instrument and BMI in the current study was clear, consistent, and broadly similar among different types of individuals. Limitations of the present study include the potential for some horizontal pleiotropy, although sensitivity analyses involving a range of alternative MR approaches yielded consistent results. Beyond mediation through diabetes, we have not yet explored other potential pathways, while the recruitment of participants aged ≥35 years means that we cannot explore the role of BMI on diseases at younger ages. The study population arises from just two districts of Mexico City and so the participants are not representative of adults throughout Mexico (or even Mexico City). However, prospective studies of non-representative cohorts of individuals can provide reliable evidence about the associations of risk factors with disease that are widely generalizable [30, 31]. Finally, a lack of information on non-fatal outcomes means that the conclusions apply directly only to causes of death.

## Conclusions

This large study of an admixed Mexican population implicates a strong causal relevance of higher genetically determined lifelong BMI with risk of death from vascular, renal, diabetic, infective, and respiratory causes, with a substantial proportion of the effect mediated through the effects of BMI on diabetes. Population-wide reductions in BMI as well as optimal diabetes control should remain key priorities for the prevention of premature death in Mexico.

## Ethics approval

Ethics approval was obtained from the Mexican Ministry of Health, the Mexican National Council of Science and Technology (reference 0595P-M), and the University of Oxford (reference C99.260). All participants provided written informed consent.

## Supplementary Material

dyaf110_Supplementary_Data

## Data Availability

Data from the MCPS are available to bona fide researchers. The study’s Data and Sample Sharing policy can be downloaded (in English or Spanish: https://www.ctsu.ox.ac.uk/research/mcps). Available study data can be examined in detail through the study’s Data Showcase, available at https://datashare.ndph.ox.ac.uk/mexico/. Statistical code: available from Prof. Emberson (jonathan.emberson@ndph.ox.ac.uk). For the purposes of open access, the authors have applied a Creative Commons Attribution (CC BY) licence to any Author Accepted Manuscript version arising.
