# Supplementary material for: A Mendelian randomization study of the effect of body mass index on 52 causes of death among 125 000 Mexican adults with admixed ancestry
Source: Int J Epidemiol. 2025 Jul 11;54(4):dyaf110. doi: 10.1093/ije/dyaf110 (PMC12254126; doi:10.1093/ije/dyaf110)
Supplement: dyaf110_Supplementary_Data [file dyaf110_supplementary_data.zip › ije-2024-10-1546-File007.pdf]

Supplementary Material

A Mendelian randomisation study of the effect of body mass index on 52 causes of death among 125 000 Mexican adults with admixed ancestry

		Pg
**Supplementary figures**		
S1.	Comparison of the effect sizes of variants associated with BMI in the trans-ancestry meta-analysis and MCPS	2
S2.	Baseline BMI in men and women by fifth of BMI-GS, overall, by age, and by proportion of Indigenous American ancestry	3
Sensitivity analyses: Association of genetically-predicted BMI with all-cause, vascular-metabolic and non vascular-metabolic mortality at ages 35-74 years, overall and by sex		
S3.	Using an alternative ‘clumping’ threshold	4
S4.	Excluding those with pre-existing diabetes or other chronic diseases from the BMI-GS to BMI association	5
S5.	Using a BMI-GS derived from GIANT	6
S6.	Scatter plots of SNP-adiposity associations against SNP-all-cause mortality associations	7
Association of genetically-predicted BMI with:		
S7.	All-cause, vascular-metabolic and non vascular-metabolic mortality across strata using the residual and the doubly-ranked method	8
S8.	Age and sex across strata using the residual and the doubly-ranked method	9
S9.	All-cause mortality at ages 35-74 years, by selected characteristics	
	a. Using sex-specific estimates of the BMI-GS to BMI association	10
	b. Using sex and subgroup-specific estimates of the BMI-GS to BMI association	11
S10.	All-cause, vascular-metabolic and non vascular-metabolic mortality at ages 75-89 years, overall and by sex	12
**Supplementary tables**		
S1.	List of ICD-10 codes contributing to each underlying cause of death at ages 35-74 years	13
S2.	Baseline characteristics of men and women aged 35-74 years, by fifths of genetically-predicted BMI	14
S3.	Sensitivity ‘two-sample’ MR analyses for all-cause mortality at ages 35-74 years, using SNP-to-BMI associations derived from a trans-ancestry GWAS of China Kadoorie Biobank and UK Biobank	18
**Supplementary methods**		19

Figure S1. Comparison of the effect sizes of variants associated with BMI in the trans−ancestry meta−analysis and MCPS

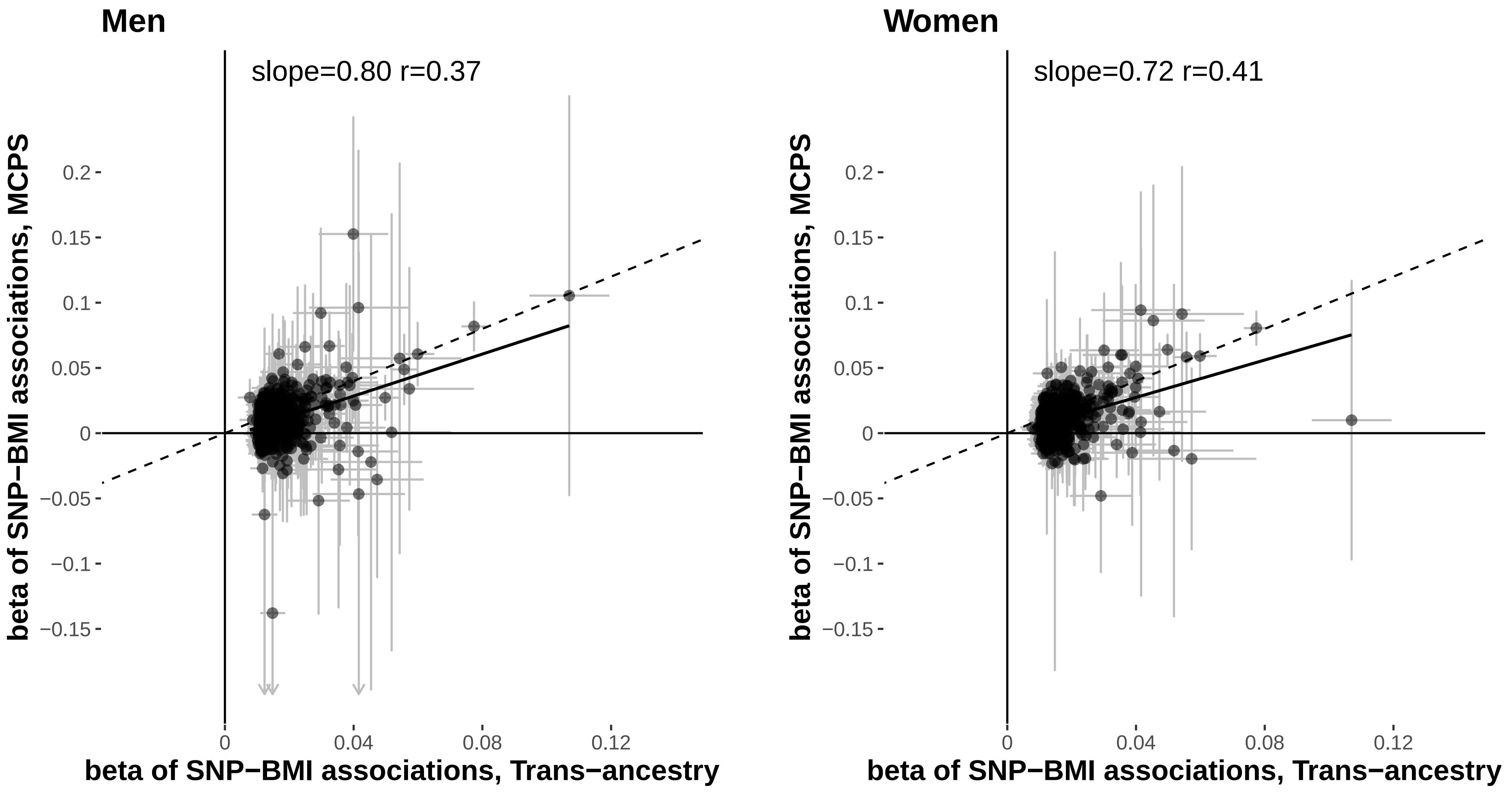

BMI was transformed by applying rank inverse normalisation to the residuals from a regression of BMI on age and sex. The beta coefficients shown are SD units of BMI in men or women respectively. The black sloped line shows the line of best fit from an inverse−variance−weighted linear regression. The dashed black line indicates the line y=x. r is the Pearson correlation coeffcient.

Figure S2: Baseline BMI in men and women by fifth of BMI−GS, overall, by age, and by proportion of Indigenous American ancestry

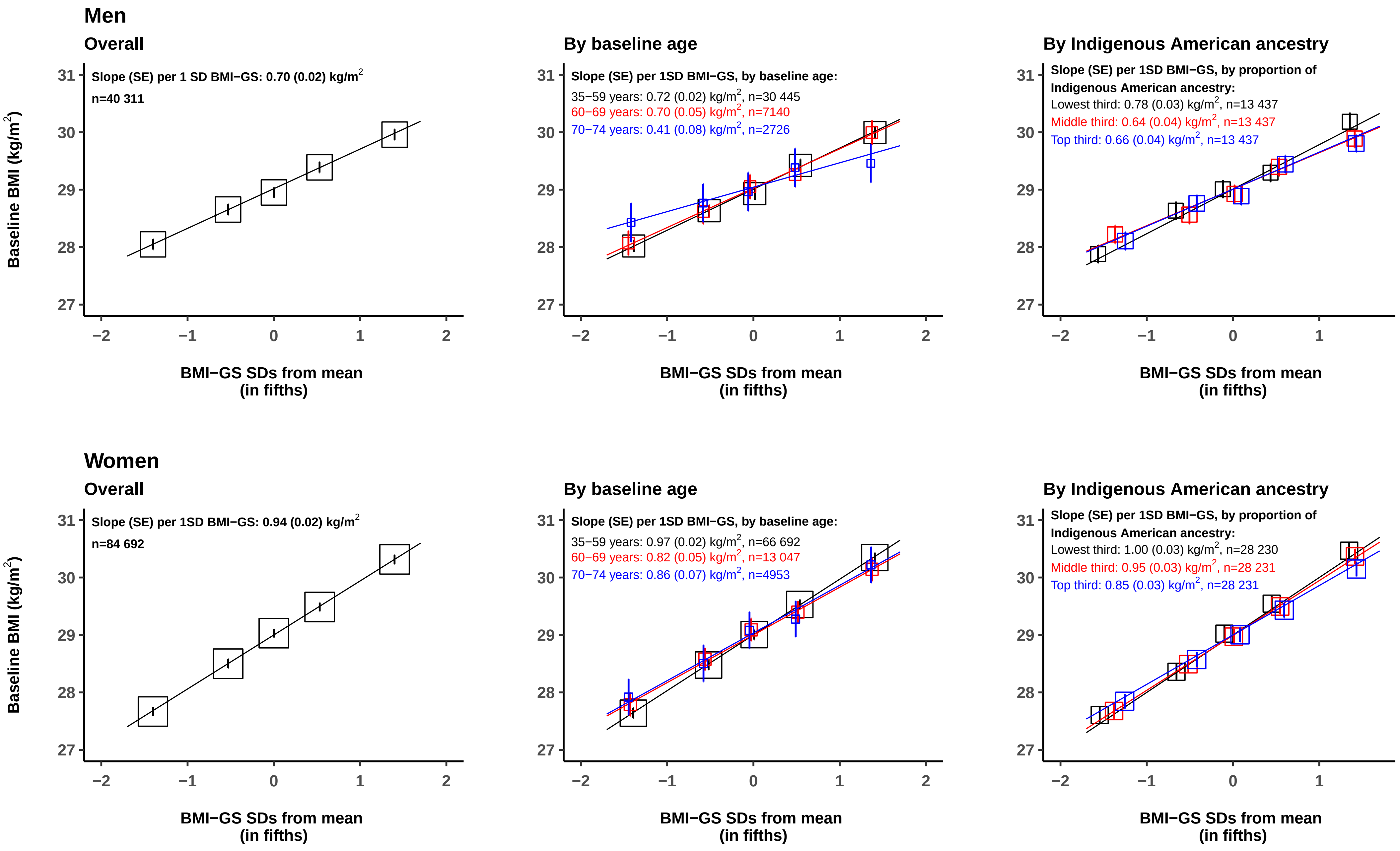

The SD of BMI−GS was 0.25 units in men (top panels) and 0.26 units in women (bottom panels). Separately for men and women, estimates are then shown per 1SD higher BMI−GS and are adjusted for age, age−squared and the first 7 genetic PCs.

Figure S3. Association of genetically-predicted BMI with all-cause, vascular-metabolic and non vascular-metabolic mortality at ages 35-74 years, overall and by sex

(Sensitivity analysis using alternative clumping threshold)

Cause of death / sex	No. deaths	HR (95% CI)
**Vascular-metabolic**		
Men	3251	2.31 (1.80, 2.97)
Women	3860	2.15 (1.82, 2.54)
**All**	**7111**	**2.20 (1.91, 2.53)**
**Non vascular-metabolic**		
Men	2411	1.59 (1.19, 2.11)
Women	3544	1.42 (1.19, 1.68)
**All**	**5955**	**1.46 (1.26, 1.69)**
**All-cause**		
Men	5662	1.97 (1.63, 2.38)
Women	7404	1.76 (1.56, 1.99)
**All**	**13066**	**1.82 (1.64, 2.01)**

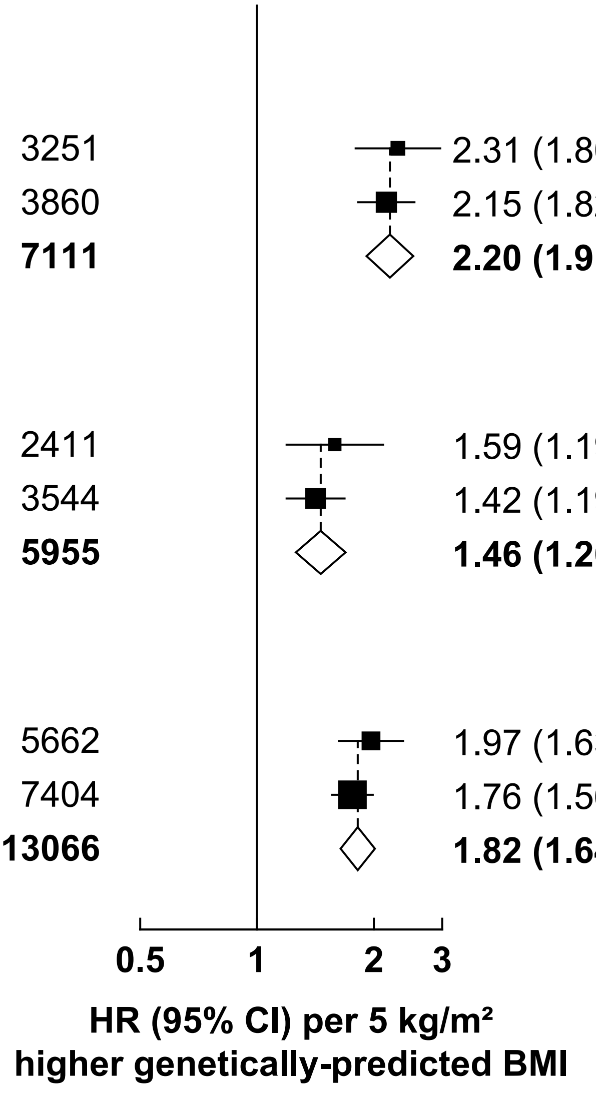

Analyses as per Figure 1, but now with weighted polygenic risk scores composed using an alternative clumping threshold of 0.05.

Figure S4. Association of genetically-predicted BMI with all-cause, vascular-metabolic and non vascular-metabolic mortality at ages 35-74 years, overall and by sex

(Sensitivity analysis excluding those with pre-existing diabetes or other chronic diseases from the BMI-GS to BMI association)

Cause of death / sex	No. deaths	HR (95% CI)
**Vascular-metabolic**		
Men	3251	2.08 (1.64, 2.64)
Women	3860	2.09 (1.77, 2.47)
**All**	**7111**	**2.09 (1.82, 2.39)**
**Non vascular-metabolic**		
Men	2411	1.56 (1.19, 2.05)
Women	3544	1.41 (1.19, 1.67)
**All**	**5955**	**1.45 (1.25, 1.68)**
**All-cause**		
Men	5662	1.84 (1.54, 2.20)
Women	7404	1.73 (1.53, 1.95)
**All**	**13066**	**1.76 (1.59, 1.95)**

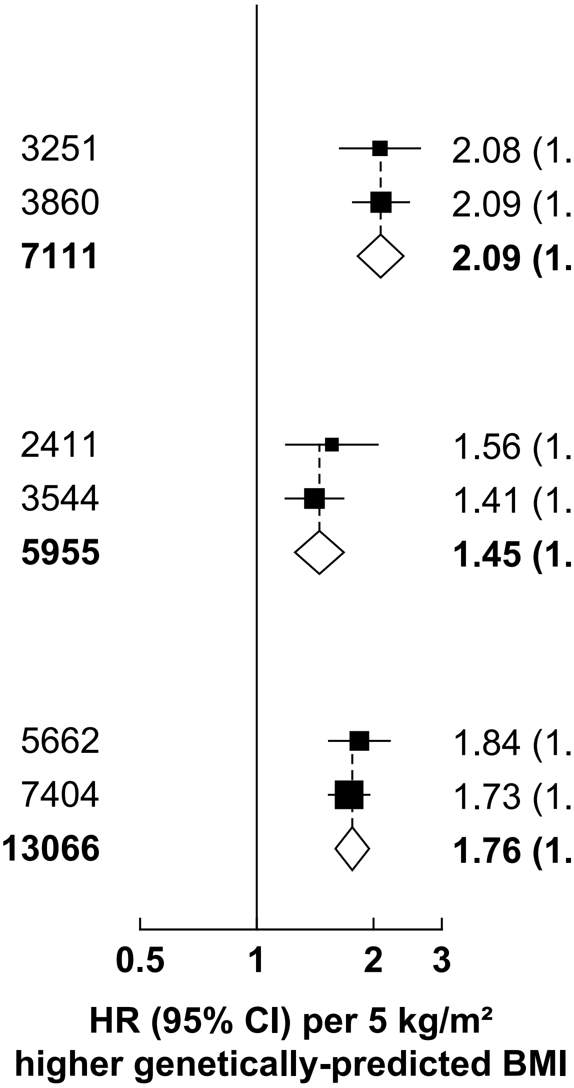

Analyses as for Figure 1, but now excluding from the GRS—BMI association those with prior diabetes or other chronic disease at recruitment.

Figure S5. Association of genetically-predicted BMI with all-cause, vascular-metabolic and non vascular-metabolic mortality at ages 35-74 years, overall and by sex

(Sensitivity analysis using a BMI-GS derived from GIANT)

**Cause of death / sex**	**No. deaths**	**HR (95% CI)**
**Vascular-metabolic**		
Men	3251	2.74 (2.10, 3.57)
Women	3860	2.73 (2.27, 3.28)
**All**	**7111**	**2.73 (2.35, 3.18)**
**Non vascular-metabolic**		
Men	2411	1.63 (1.21, 2.20)
Women	3544	1.52 (1.26, 1.84)
**All**	**5955**	**1.55 (1.32, 1.82)**
**All-cause**		
Men	5662	2.19 (1.79, 2.68)
Women	7404	2.06 (1.81, 2.35)
**All**	**13066**	**2.10 (1.88, 2.35)**

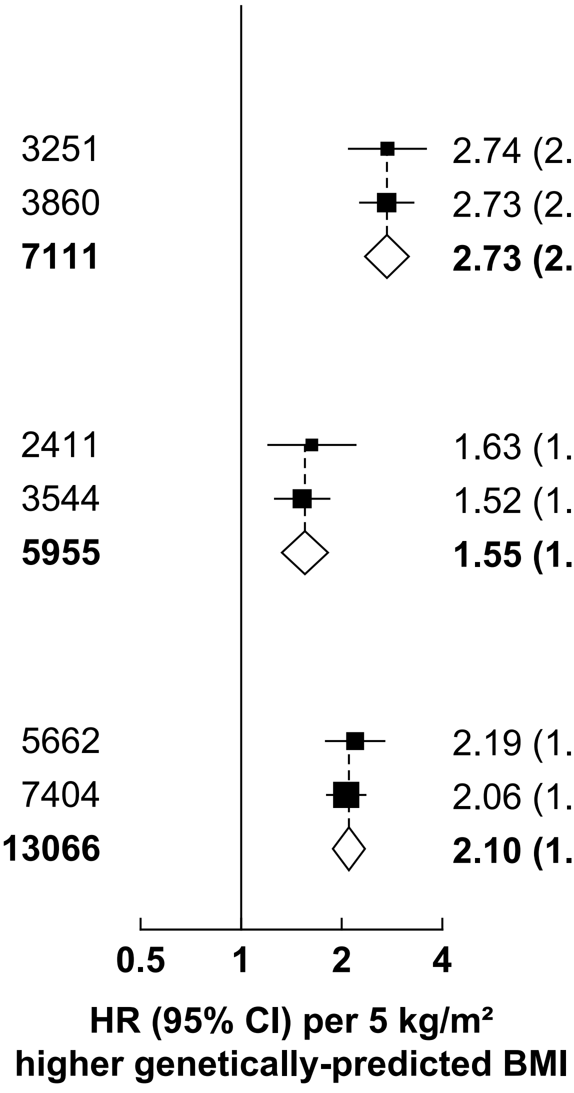

Analyses as per Figure 1, but now with an alternative weighted polygenic risk score omposed using SNPs from the GIANT Consortia.

Figure S6. Scatter plots of SNP−adiposity associations against SNP−all−cause mortality associations

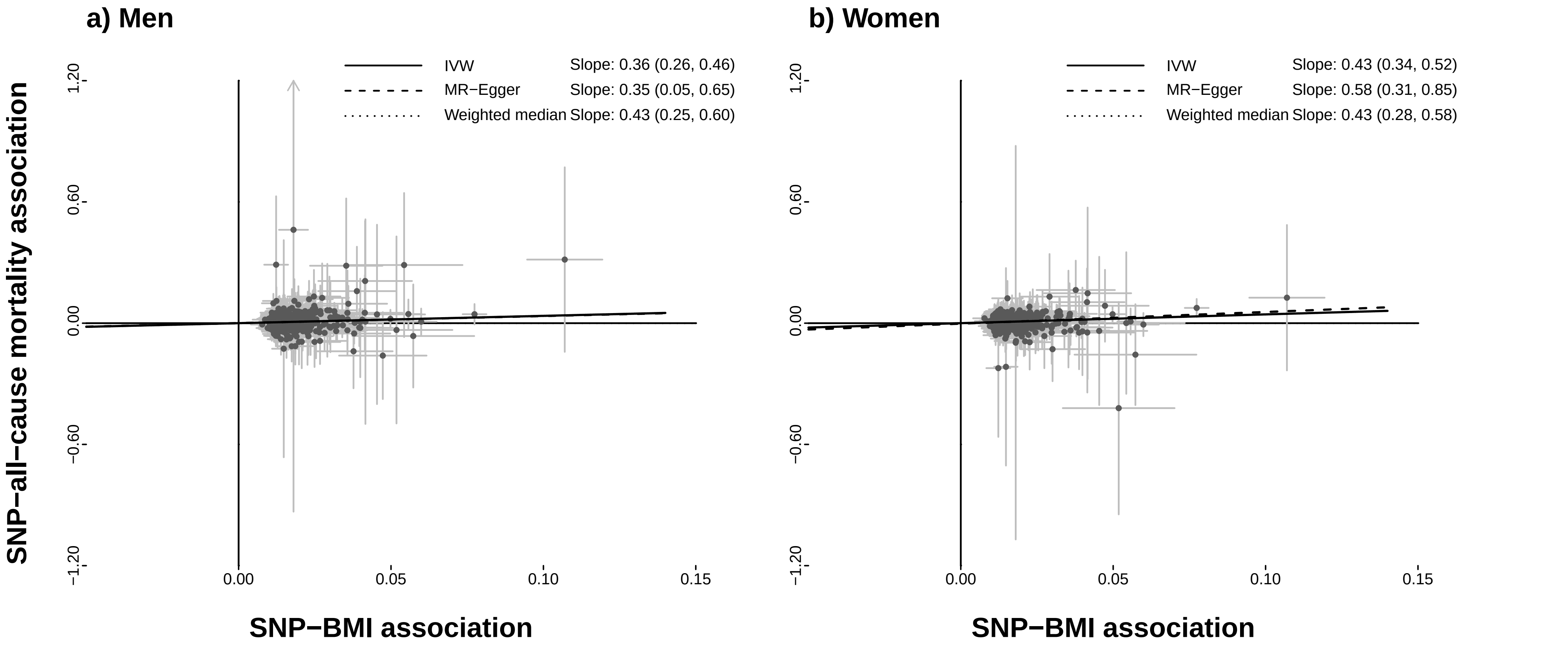

IVW and MR−Egger slopes calculated using iterative weights in a multiplicative random effects model. For each approach the SNP−BMI effect estimates (per 1 SD BMI) and their standard errors are taken from trans−ancestry meta−analysis of China Kadoorie Biobank with UK Biobank, while the SNP−mortality effect estimates (lnHR) and their standard errors are taken from MCPS.

Figure S7. All−cause, vascular−metabolic and non vascular−metabolic mortality across strata using the residual and the doubly−ranked method

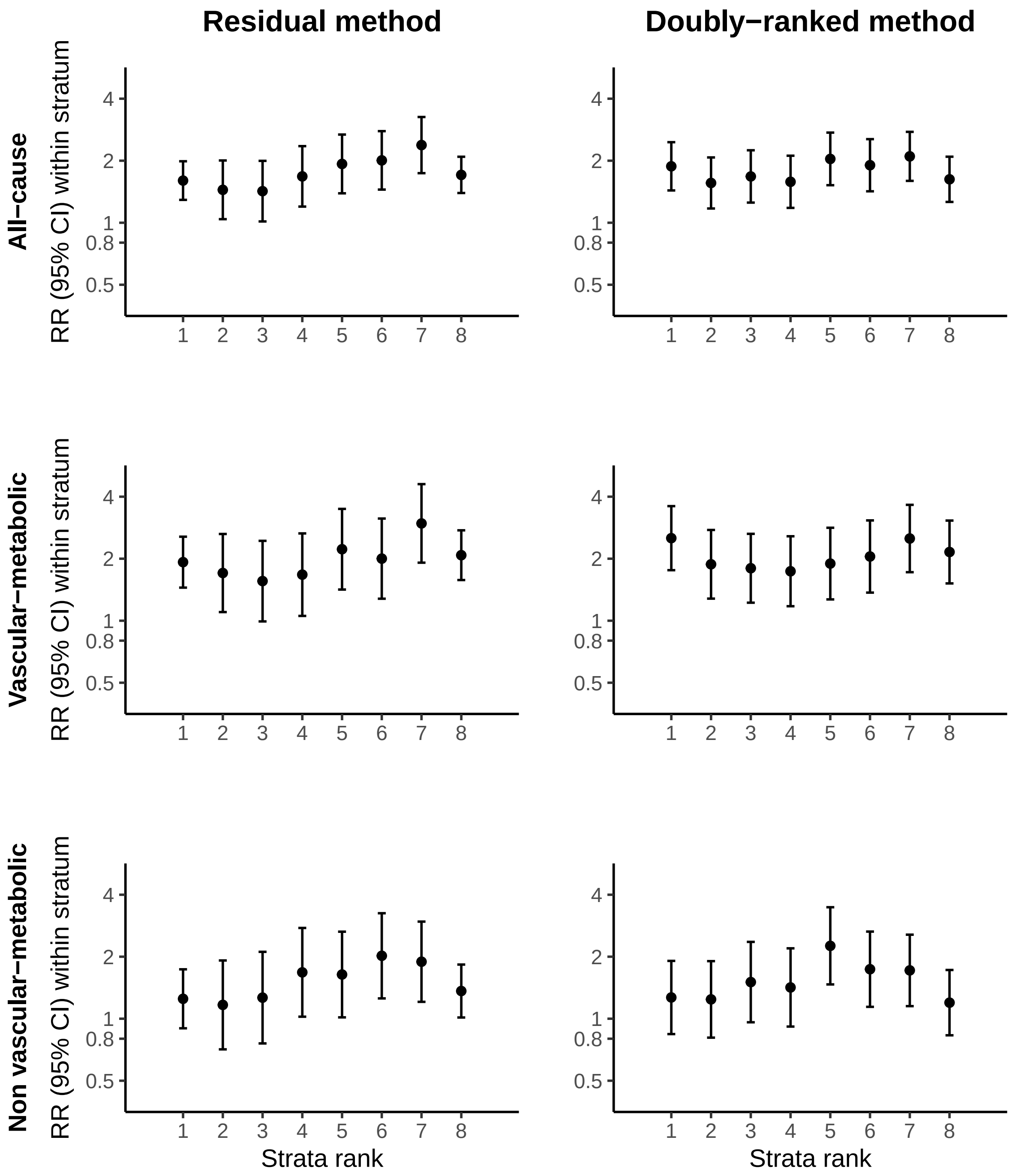

For the 'residual' method, 8 equally−sized groups are created from the residuals after regressing BMI on BMI−GS. For the 'doubly−ranked' method, participants are first split into 8 equally−sized groups on the basis of their BMI−GS (the 'first strata'). Then within each of these 8 groups, participants are split into 8 additional groups on the basis of their BMI (the 'second strata'). Each of the second strata levels are then combined across levels of the first strata. For example, the first group shown above includes those with the lowest BMI in BMI−GS strata 1, the lowest BMI in BMI−GS strata 2, and so on, up to and including the lowest BMI in BMI−GS strata 8. For both methods, the MR ratio method is then used to estimate a hazard ratio and 95% CI in each group, in which the BMI−GS to mortality association is estimated only for that group, but the BMI−GS to BMI association is estimated in all participants. Three participants were randomly dropped from the analysis to obtain equally−sized strata.

Figure S8. Age and sex across strata using the residual and the doubly−ranked method

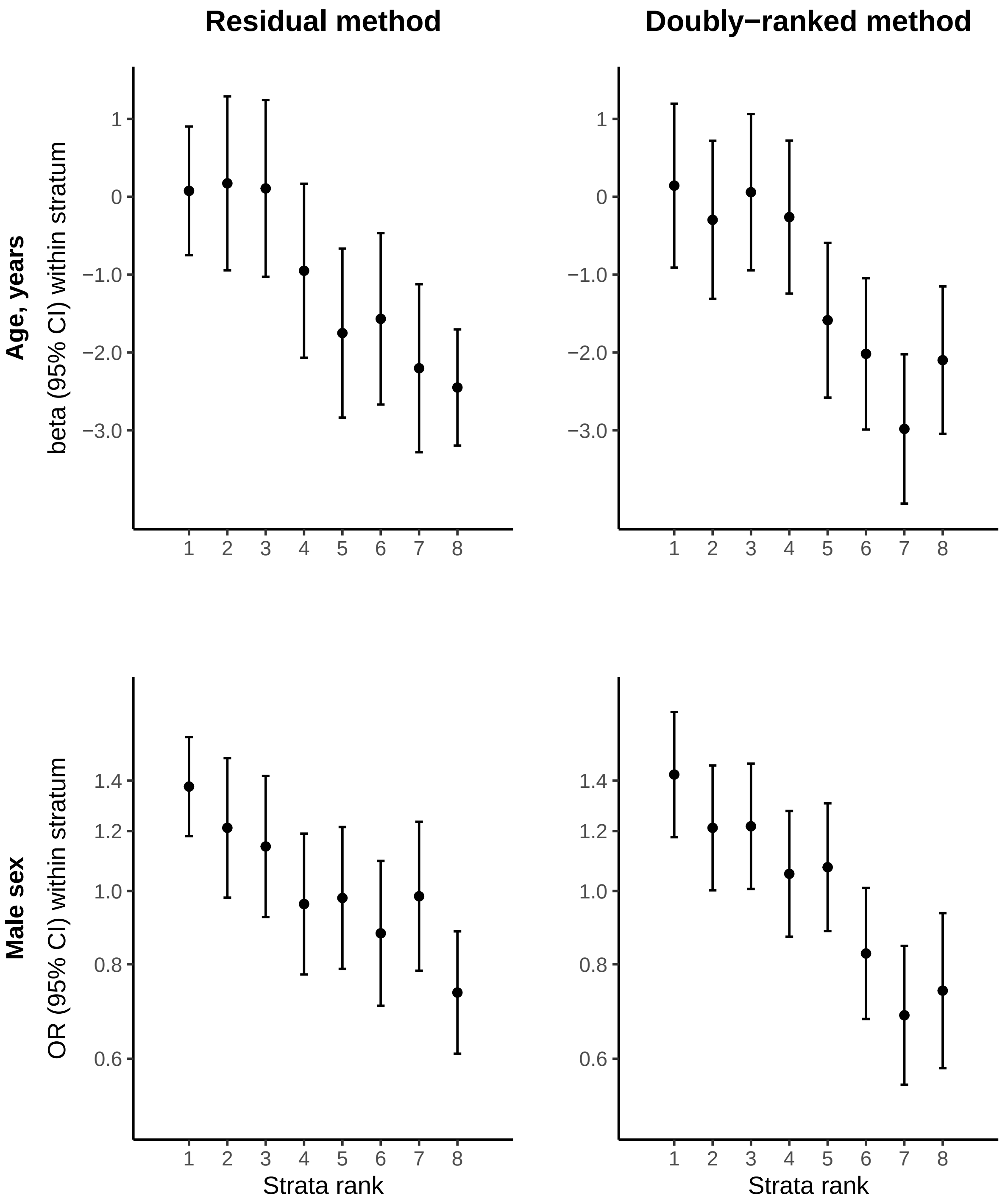

For the 'residual' method, 8 equally−sized groups are created from the residuals after regressing BMI on BMI−GS. For the 'doubly−ranked' method, participants are first split into 8 equally−sized groups on the basis of their BMI−GS (the 'first strata'). Then within each of these 8 groups, participants are split into 8 additional groups on the basis of their BMI (the 'second strata'). Each of the second strata levels are then combined across levels of the first strata. For example, the first group shown above includes those with the lowest BMI in BMI−GS strata 1, the lowest BMI in BMI−GS strata 2, and so on, up to and including the lowest BMI in BMI−GS strata 8. For both methods, the MR ratio method is then used to estimate an estimate and 95% CI in each group, in which the BMI−GS to age/sex association is estimated only for that group, but the BMI−GS to BMI association is estimated in all participants. Three participants were randomly dropped from the analysis to obtain equally−sized strata.

Figure S9a. Association of genetically-predicted BMI with all-cause mortality at ages 35-74 years, by selected characteristics

(sex-specific estimates of the BMI-GS to BMI association)

	No. deaths	HR (95% CI)
**Age at risk**		
35-59 yrs	4000	2.43 (2.01, 2.93)
60-69 yrs	5563	1.71 (1.46, 2.00)
70-74 yrs	3503	1.43 (1.17, 1.74)
		Heterogeneity: χ^2_2=15.4 (p<0.0005)
		Trend: χ^2_1=14.7 (p<0.001)
**Residential District**		
Coyoacan	4312	1.58 (1.32, 1.90)
Iztapalapa	8754	1.90 (1.67, 2.15)
		Heterogeneity: χ^2_1=2.6 (p=0.11)
**Indigenous Ancestry**		
Bottom third	3738	1.57 (1.30, 1.89)
Middle third	4375	1.84 (1.54, 2.19)
Top third	4953	2.01 (1.69, 2.39)
		Heterogeneity: χ^2_2=3.7 (p=0.16)
		Trend: χ^2_1=3.6 (p=0.06)
**Leisure-time physical activity**		
None	10574	1.77 (1.58, 1.98)
At least 1 day per week	2492	1.90 (1.49, 2.43)
		Heterogeneity: χ^2_1=0.3 (p=0.6)
**Smoking**		
Never	5658	1.82 (1.57, 2.11)
Former	2799	1.82 (1.45, 2.30)
Current	4598	1.71 (1.42, 2.06)
		Heterogeneity: χ^2_2=0.3 (p=0.86)
		Trend: χ^2_1=0.25 (p=0.62)
**All**	**13066**	**1.80 (1.63, 2.00)**

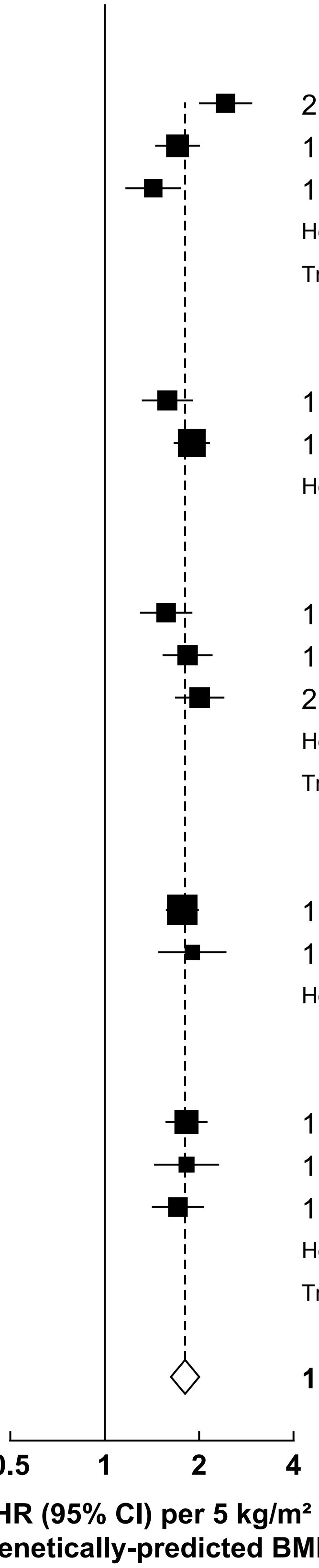

Figure S9b. Association of genetically-predicted BMI with all-cause mortality at ages 35-74 years, by selected characteristics

(sex and subgroup specific estimates of the BMI-GS to BMI association)

	No. deaths	HR (95% CI)
**Age at risk**		
35-59 yrs	4000	2.36 (1.97, 2.84)
60-69 yrs	5563	1.71 (1.46, 2.01)
70-74 yrs	3503	1.47 (1.19, 1.82)
		Heterogeneity: χ^2_2=12.2 (p=0.002)
		Trend: χ^2_1=11.6 (p<0.001)
**Residential District**		
Coyoacan	4312	1.57 (1.32, 1.88)
Iztapalapa	8754	1.92 (1.68, 2.19)
		Heterogeneity: χ^2_1=3.2 (p=0.08)
**Indigenous Ancestry**		
Bottom third	3738	1.53 (1.28, 1.82)
Middle third	4375	1.84 (1.53, 2.20)
Top third	4953	2.13 (1.76, 2.59)
		Heterogeneity: χ^2_2=6.5 (p=0.04)
		Trend: χ^2_1=6.4 (p=0.01)
**Leisure-time physical activity**		
None	10574	1.75 (1.56, 1.95)
At least 1 day per week	2492	2.05 (1.56, 2.70)
		Heterogeneity: χ^2_1=1.1 (p=0.29)
**Smoking**		
Never	5658	1.86 (1.59, 2.16)
Former	2799	1.77 (1.41, 2.21)
Current	4598	1.69 (1.41, 2.03)
		Heterogeneity: χ^2_2=0.6 (p=0.74)
		Trend: χ^2_1=0.6 (p=0.44)
**All**	**13066**	**1.80 (1.63, 2.00)**

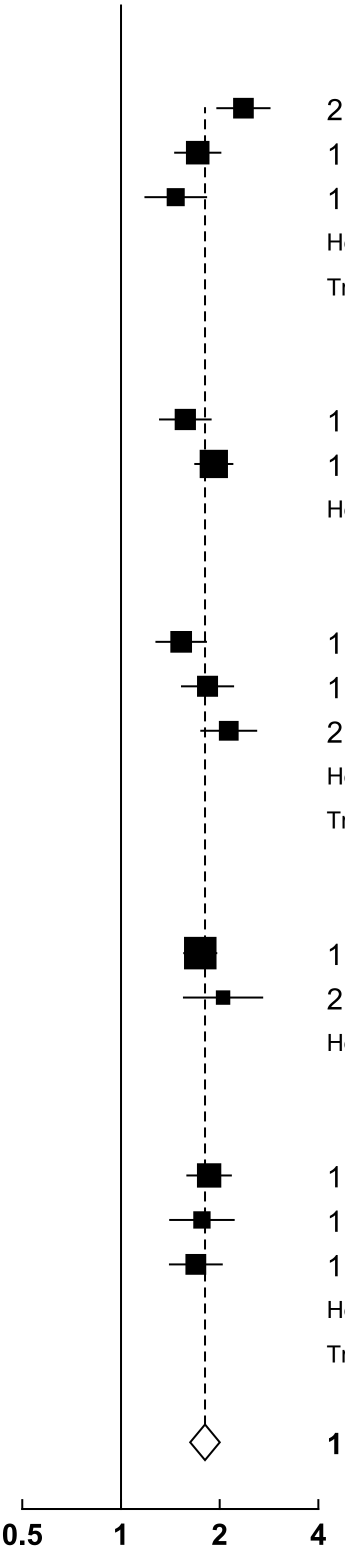

HR (95% CI) per 5 kg/m² genetically-predicted BMI

Figure S10. Association of genetically-predicted BMI with all-cause, vascular-metabolic and non vascular-metabolic mortality at ages 75-89 years, overall and by sex

Cause of death / sex	No. deaths	HR (95% CI)
**Vascular-metabolic**		
Men	2291	1.23 (0.88, 1.73)
Women	3345	1.54 (1.25, 1.90)
**All**	**5636**	**1.45 (1.22, 1.73)**
**Non vascular-metabolic**		
Men	2026	1.32 (0.92, 1.88)
Women	2439	1.34 (1.05, 1.70)
**All**	**4465**	**1.33 (1.09, 1.62)**
**All-cause**		
Men	4317	1.27 (0.99, 1.62)
Women	5784	1.45 (1.24, 1.70)
**All**	**10101**	**1.40 (1.22, 1.60)**

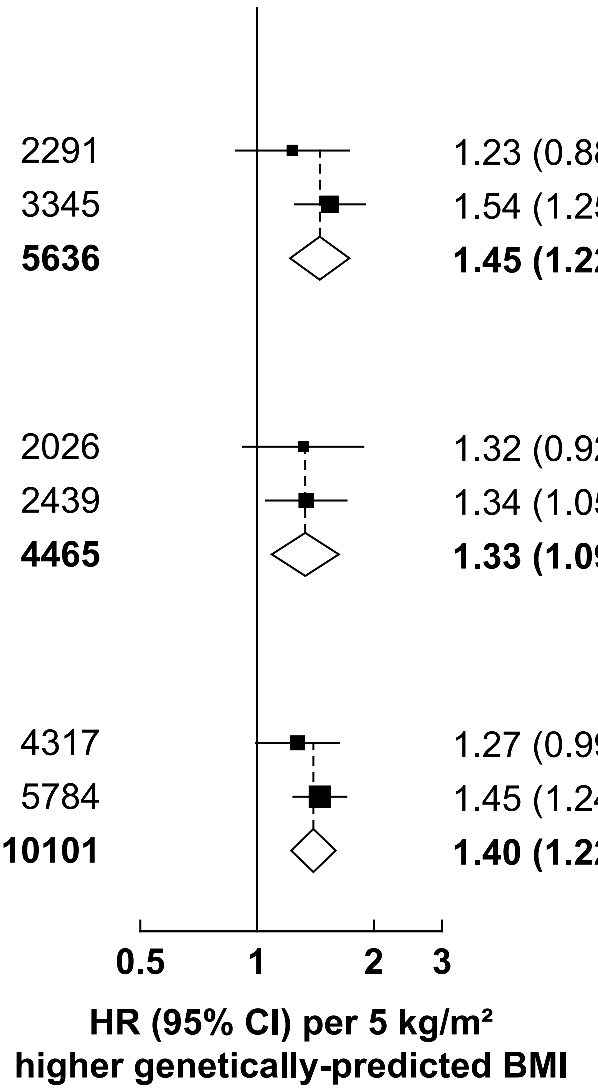

Table S1: Baseline characteristics of men and women aged 35-74 years, by fifths of genetically predicted BMI

	Fifths of genetically predicted BMI in men					Fifths of genetically predicted BMI in women				
	I N=8063	II N=8062	III N=8062	IV N=8062	V N=8062	I N=16939	II N=16938	III N=16939	IV N=16938	V N=16938
Age, years	51 (11)	51 (11)	51 (11)	51 (11)	50 (11)	50 (11)	50 (11)	50 (11)	50 (11)	50 (11)
Indigenous ancestry % (median, IQR)	65% (52%-79%)	67% (54%-81%)	67% (54%-81%)	67% (54%-82%)	67% (54%-80%)	67% (54%-81%)	68% (55%-82%)	68% (55%-82%)	67% (55%-82%)	67% (54%-79%)
**Sociodemographic status and lifestyle characteristics**										
Resident of Coyoacán	42%	43%	41%	41%	42%	39%	37%	37%	36%	36%
University/college educated	26%	25%	24%	25%	24%	12%	12%	12%	11%	12%
Current smoker	49%	50%	51%	52%	53%	23%	23%	24%	24%	25%
Current drinker	78%	78%	78%	77%	77%	63%	63%	63%	63%	63%
Regular leisure-time physical activity	32%	30%	31%	29%	30%	19%	19%	19%	18%	18%
**Physical measurements**										
Height, cm	165 (7.0)	165 (6.9)	165 (7.1)	165 (7.0)	165 (6.9)	152 (6.4)	152 (6.4)	152 (6.3)	152 (6.2)	152 (6.2)
Weight, kg	74 (12.2)	75 (12.4)	76 (12.7)	77 (12.6)	79 (13.6)	65 (11.7)	67 (12.3)	68 (12.5)	69 (12.8)	71 (13.3)
BMI, kg/m^2	27.1 (4.0)	27.6 (4.1)	28.1 (4.2)	28.4 (4.2)	29.0 (4.5)	28.4 (4.8)	29.2 (4.9)	29.7 (5.1)	30.2 (5.2)	31.0 (5.4)
Waist-hip-ratio	0.95 (0.07)	0.95 (0.07)	0.95 (0.06)	0.95 (0.06)	0.96 (0.07)	0.87 (0.07)	0.87 (0.07)	0.88 (0.07)	0.88 (0.07)	0.88 (0.06)
Systolic blood pressure, mmHg	127 (15)	128 (16)	128 (15)	129 (15)	129 (15)	125 (16)	126 (17)	126 (17)	127 (17)	127 (17)
Diastolic blood pressure, mmHg	84 (10)	84 (10)	84 (10)	85 (10)	85 (10)	82 (10)	82 (10)	82 (10)	83 (10)	83 (10)
**Diabetes**										
Previously-diagnosed diabetes[a]	11%	12%	14%	14%	15%	11%	12%	13%	15%	15%
Undiagnosed diabetes[b]	5%	5%	5%	6%	6%	5%	5%	5%	5%	6%
Subtotal: any diabetes	16%	17%	19%	20%	21%	16%	17%	18%	20%	21%
HbA1c in those without diabetes, %	5.4 (5.2-5.6)	5.5 (5.3-5.7)	5.5 (5.3-5.7)	5.5 (5.3-5.7)	5.5 (5.3-5.7)	5.4 (5.2-5.7)	5.5 (5.2-5.7)	5.5 (5.2-5.7)	5.5 (5.3-5.7)	5.5 (5.2-5.7)
HbA1c in those with diabetes, %	8.8 (6.8-10.5)	8.9 (6.8-10.6)	9.0 (6.8-10.8)	8.8 (6.8-10.5)	9.0 (6.9-10.8)	8.8 (6.8-10.6)	8.9 (6.8-10.8)	8.9 (6.9-10.8)	9.1 (6.9 -10.9)	9.1 (6.9-11.0)
**Other chronic disease and medication use**										
Other chronic disease[c]	4%	4%	4%	5%	5%	4%	4%	4%	5%	5%
Any antihypertensive	10%	10%	11%	11%	11%	15%	15%	16%	17%	18%
Any antithrombotic	3%	2%	3%	3%	3%	3%	3%	3%	3%	3%
Any lipid-lowering	1%	1%	1%	1%	1%	1%	<1%	<1%	1%	1%

Data are %, mean (SD) or median (IQR). HbA1c=Glycosylated haemoglobin. BMI= Body Mass Index. a Self-reported diagnosis or taking a diabetes medication. b No previously-diagnosed diabetes but HbA1c ≥6.5%. c Cardiovascular disease, stroke, chronic kidney disease, liver cirrhosis, cancer, or chronic obstructive pulmonary disease.

Table S2: List of ICD-10 codes contributing to each underlying cause of death at ages 35-74 years

Cause of death	ICD-10 codes (and number of deaths)
Acute myocardial Infarction (n=1800)	I210 (20), I211 (12), I213 (1), I219 (1763), I220 (2), I221 (1), I229 (1)
Chronic ischaemic heart disease (n=189)	I200 (3), I209 (3), I249 (32), I251 (47), I252 (1), I258 (9), I259 (94)
Heart failure (n=249)	I099 (16), I110 (99), I500 (52), I501 (11), I509 (71)
Other cardiac death (n=201)	I011 (1), I018 (1), I050 (2), I051 (1), I059 (18), I069 (2), I070 (1), I071 (1), I079 (1), I080 (3), I081 (1), I091 (1), I119 (11), I270 (4), I272 (3), I279 (7), I319 (1), I330 (7), I340 (3), I350 (10), I351 (1), I358 (2), I38X (8), I420 (10), I421 (1), I426 (1), I429 (1), I442 (9), I443 (2), I460 (1), I469 (7), I471 (2), I472 (3), I489 (3), I48X (7), I490 (6), I499 (6), I515 (2), I518 (3), I519 (9), Q210 (1), Q213 (1), Q238 (1), Q248 (1), R570 (34)
**Subtotal: Cardiac (n=2439)**	**Any of the above four categories**
Ischaemic stroke (n=88)	I633 (4), I634 (18), I635 (3), I638 (1), I639 (54), I693 (8)
Haemorrhagic stroke (n=320)	I600 (2), I602 (1), I608 (1), I609 (76), I61 (1), I610 (1), I612 (1), I613 (1), I614 (1), I615 (2), I618 (1), I619 (220), I620 (6), I629 (4), I690 (1), I691 (1)
Other cerebrovascular disease (n=333)	F019 (2), I669 (6), I671 (5), I672 (2), I673 (1), I674 (3), I678 (80), I679 (105), I694 (6), I698 (29), I64X (94)
**Subtotal: Stroke (n=741)**	**Any of the above three categories**
Thromboembolism (n=192)	E115 (36), E145 (27), I260 (2), I269 (78), I710 (4), I712 (1), I713 (5), I718 (3), I719 (1), I729 (2), I731 (1), I739 (3), I740 (1), I741 (1), I743 (1), I771 (15), I776 (1), I779 (1), I802 (5), I803 (1), I822 (1), I829 (2)
Other vascular (n=79)	I839 (1), I872 (5), I879 (1), I890 (1), I99X (4), K550 (59), K551 (1), K552 (1), K559 (5), K761 (1)
**Subtotal: Vascular (n=3451)**	**Any cardiac, cerebrovascular or other vascular categories**
Chronic (or unspecified) kidney disease (n=1724)	E102 (9), E112 (854), E142 (332), I120 (107), I129 (1), I130 (3), I131 (2), I132 (27), N039 (25), N049 (2), N059 (11), N12X (12), N142 (1), N180 (12), N185 (32), N189 (225), N19X (62), N281 (1), N289 (5), Y841 (1)
Acute kidney injury (n=83)	N002 (1), N009 (8), N179 (74)
Other urinary tract (incl. infections) (n=227)	N10X (2), N151 (12), N200 (6), N201 (1), N300 (1), N309 (1), N390 (204)
**Subtotal: Renal (n=2034)**	**Any of the above three categories**
**Acute diabetic crises (n=557)**	E100 (3), E101 (2), E110 (166), E111 (187), E140 (104), E141 (88), E162 (7)

Table S2: List of ICD-10 codes contributing to each underlying cause of death at ages 35-74 years

Cause of death	ICD-10 codes (and number of deaths)
Liver cirrhosis (n=922)	B169 (3), B171 (28), B182 (12), B189 (1), B190 (2), B199 (2), I850 (16), I859 (6), K701 (15), K702 (1), K703 (162), K704 (23), K709 (16), K711 (2), K716 (1), K720 (9), K721 (58), K729 (206), K739 (2), K742 (1), K743 (2), K745 (2), K746 (287), K750 (14), K754 (4), K759 (2), K764 (1), K766 (11), K767 (14), K768 (1), K769 (17), Q447 (1)
Gallstone, biliary or pancreatic (n=147)	D136 (1), K800 (4), K801 (9), K802 (4), K803 (3), K804 (1), K805 (2), K810 (12), K811 (3), K819 (5), K821 (1), K822 (2), K829 (4), K830 (17), K831 (3), K839 (1), K851 (1), K852 (4), K858 (7), K859 (33), K85X (24), K861 (3), K868 (3)
**Subtotal: Hepatobiliary (n=1069)**	**Either of the above two categories**
**Cancer**	
Upper aero-digestive (n=38)	C029 (12), C07X (2), C089 (1), C109 (3), C119 (1), C139 (1), C140 (5), C142 (1), C329 (12)
Lung (n=152)	C33X (1), C349 (140), D380 (2), D381 (7), D383 (2)
Oesophageal/stomach (n=226)	C159 (16), C160 (3), C169 (201), D371 (6)
Pancreatic (n=111)	C250 (21), C252 (1), C259 (89)
Hepatobiliary (n=246)	C220 (49), C221 (24), C229 (105), C23X (24), C240 (12), C241 (9), C248 (3), C249 (14), D376 (6)
Other gastrointestinal (n=162)	C170 (10), C179 (1), C182 (3), C184 (1), C187 (2), C189 (112), C19X (4), C20X (21), C211 (1), D372 (1), D374 (6)
Breast (n=231)	C509 (230), D486 (1)
Endometrial/Uterine (n=37)	C541 (24), C549 (2), C55X (11)
Cervical (n=114)	C530 (1), C539 (113)
Other gynaecological (not uterus/cervix) (n=111)	C519 (3), C52X (2), C56X (101), D391 (4), D397 (1)
Urinary tract (n=114)	C64X (84), C679 (24), C680 (2), C689 (1), D410 (3)
Prostate (n=74)	C61X (74)
Haematological (n=187)	C817 (1), C819 (9), C822 (1), C829 (1), C830 (1), C833 (13), C838 (1), C839 (3), C844 (1), C845 (3), C851 (1), C857 (2), C859 (43), C900 (40), C901 (1), C902 (1), C910 (20), C911 (2), C920 (26), C921 (8), C927 (2), C929 (3), C950 (2), C959 (2)
Other cancer (n=202)	C319 (1), C37X (1), C382 (1), C383 (1), C384 (1), C412 (2), C419 (7), C435 (1), C437 (1), C438 (1), C439 (10), C444 (1), C445 (1), C447 (1), C449 (7), C451 (2), C457 (1), C459 (4), C469 (1), C479 (1), C480 (8), C482 (4), C492 (4), C495 (1), C499 (14), C609 (2), C629 (1), C694 (1), C709 (2), C710 (12), C711 (1), C718 (2), C719 (43), C720 (1), C729 (1), C73X (28), C741 (1), C749 (1), C97X (1), D377 (2), D429 (1), D430 (14), D432 (1), D449 (1), D487 (9)
Ill-defined (n=102)	C260 (3), C269 (2), C760 (6), C761 (1), C762 (3), C763 (2), C764 (1), C765 (2), C780 (7), C786 (5), C787 (7), C788 (2), C793 (3), C794 (3), C795 (1), C796 (1), C798 (3), C800 (27), C809 (18), C80X (2), D489 (3)
**Subtotal: Cancer (n=2107)**	**Any of the above fifteen categories**

Table S2: List of ICD-10 codes contributing to each underlying cause of death at ages 35-74 years

Cause of death	ICD-10 codes (and number of deaths)
Chronic obstructive pulmonary disease (n=310)	J42X (15), J439 (29), J440 (95), J441 (2), J448 (6), J449 (163)
Pneumonia (n=714)	J129 (4), J151 (3), J157 (2), J159 (35), J180 (54), J181 (27), J182 (2), J189 (587)
COVID-19 (n=801)	U071 (504), U072 (296), U099 (1)
Other respiratory (n=225)	A162 (8), A165 (1), A169 (2), B206 (2), B440 (1), B441 (1), B909 (1), E848 (1), J069 (1), J09 (3), J09X (1), J100 (2), J110 (1), J111 (1), J209 (4), J22X (17), J348 (1), J391 (1), J459 (9), J46X (2), J47X (2), J60X (1), J64X (4), J677 (1), J679 (2), J680 (1), J690 (3), J80X (4), J81X (2), J841 (72), J848 (1), J849 (10), J850 (2), J852 (2), J869 (9), J90X (5), J939 (1), J942 (2), J960 (5), J961 (1), J969 (3), J980 (1), J984 (16), J985 (5), J988 (8), J989 (1), Q311 (1)
**Subtotal: Respiratory (n=2050)**	**Any of the above four categories**
Septicaemia (n=197)	A415 (1), A419 (191), A483 (1), R572 (4)
Gastro-intestinal infections, peptic ulcer (n=409)	A047 (3), A060 (1), A090 (19), A099 (33), A09X (11), B462 (1), B690 (2), K052 (1), K223 (2), K228 (1), K251 (3), K254 (10), K255 (11), K256 (5), K259 (6), K264 (5), K265 (3), K269 (2), K274 (3), K275 (1), K290 (6), K291 (1), K292 (1), K295 (5), K297 (1), K318 (4), K352 (4), K353 (4), K358 (5), K359 (2), K37X (1), K572 (2), K578 (5), K579 (13), K610 (3), K612 (1), K650 (29), K658 (1), K659 (55), K920 (27), K921 (4), K922 (112)
Skin/bone/connective tissue infections (n=127)	A480 (1), L021 (4), L022 (4), L023 (2), L024 (2), L031 (5), L038 (1), L039 (3), L089 (39), L899 (7), L89X (4), L984 (6), M009 (1), M600 (2), M725 (1), M726 (19), M798 (23), M869 (3)
Other Infections (n=78)	A170 (1), A181 (2), A182 (1), A183 (1), A199 (3), A498 (1), A810 (2), A86X (2), B200 (1), B201 (1), B207 (4), B208 (7), B210 (2), B212 (1), B218 (1), B227 (3), B238 (4), B24X (4), B259 (1), B373 (1), B451 (1), B465 (2), B948 (2), B99X (1), G009 (3), G039 (4), G042 (1), G049 (7), G060 (1), H440 (1), H664 (1), H669 (1), N410 (1), N419 (1), N498 (6), N719 (1), N739 (1)
**Subtotal: Infections (excluding renal and respiratory) (n=811)**	**Any of the above four categories**

Table S2: List of ICD-10 codes contributing to each underlying cause of death at ages 35-74 years

Cause of death	ICD-10 codes (and number of deaths)
Ill-defined*/other/ external (excluding respiratory non-infective) (n=987)	D033 (1), D27X (1), D320 (1), D329 (9), D352 (1), D420 (1), D467 (1), D469 (6), D471 (1), D472 (1), D474 (1), D479 (1), D619 (6), D649 (5), D682 (1), D693 (2), D694 (1), D696 (3), D699 (3), D70X (2), D733 (1), D762 (1), E035 (1), E039 (13), E049 (1), E055 (1), E059 (3), E065 (1), E116 (3), E119 (8), E129 (1), E146 (3), E149 (5), E230 (1), E249 (2), E279 (1), E43X (1), E440 (1), E660 (1), E725 (1), E86X (4), E870 (1), E872 (9), E874 (1), E875 (2), E876 (1), E878 (1), E889 (2), F03X (6), F09X (1), F102 (5), F182 (1), F209 (2), G10X (6), G121 (1), G122 (17), G20X (6), G231 (1), G300 (1), G309 (1), G310 (1), G35X (1), G379 (1), G403 (1), G409 (10), G419 (2), G439 (1), G589 (1), G610 (5), G709 (1), G710 (2), G809 (1), G822 (1), G919 (4), G931 (9), G934 (5), G935 (1), G936 (2), G938 (1), G958 (1), I10X (3), K088 (1), K102 (1), K137 (1), K389 (1), K403 (1), K404 (1), K409 (1), K419 (1), K420 (4), K429 (2), K430 (2), K439 (2), K440 (1), K460 (3), K461 (1), K469 (2), K513 (1), K519 (1), K529 (6), K560 (2), K562 (1), K566 (40), K593 (5), K628 (1), K630 (1), K631 (23), K632 (4), K635 (1), K638 (1), K639 (5), K661 (1), K918 (1), K931 (1), L100 (1), L109 (1), L511 (2), L512 (1), L921 (1), L958 (1), L988 (1), M050 (1), M068 (1), M069 (16), M100 (1), M109 (1), M139 (1), M311 (2), M313 (1), M319 (1), M321 (3), M329 (1), M340 (1), M349 (1), M469 (1), M623 (6), M799 (1), N40X (8), N948 (1), O720 (1), R040 (1), R100 (3), R11X (1), R190 (1), R568 (1), R571 (17), R579 (2), R58X (3), R688 (20), R69X (1), R99X (184), S729 (2), T07X (1), T874 (1), V011 (1), V029 (1), V041 (1), V051 (1), V093 (3), V099 (65), V182 (1), V209 (1), V299 (1), V439 (1), V489 (1), V496 (1), V499 (12), V580 (1), V719 (1), V785 (1), V878 (6), V892 (10), V899 (3), W018 (1), W100 (13), W104 (1), W105 (1), W108 (2), W126 (1), W130 (9), W134 (2), W138 (1), W139 (2), W170 (4), W172 (1), W174 (1), W176 (1), W178 (2), W179 (1), W18 (1), W180 (5), W181 (1), W184 (2), W188 (1), W190 (8), W194 (1), W195 (1), W199 (3), W206 (1), W250 (1), W314 (1), W340 (1), W370 (1), W744 (2), W748 (3), W769 (1), W780 (1), W789 (1), W799 (1), W849 (1), W871 (1), W878 (1), X09 (1), X090 (3), X094 (1), X099 (1), X219 (1), X314 (1), X360 (1), X459 (1), X470 (1), X590 (3), X594 (1), X598 (1), X599 (26), X640 (1), X650 (1), X680 (1), X700 (5), X740 (3), X780 (3), X800 (1), X840 (1), X910 (1), X914 (2), X950 (3), X954 (18), X955 (1), X959 (1), X990 (6), X994 (5), X999 (2), Y044 (1), Y048 (1), Y084 (1), Y099 (1), Y159 (1), Y200 (1), Y240 (1), Y244 (3), Y245 (1), Y248 (1), Y249 (2), Y260 (2), Y280 (1), Y330 (1), Y334 (1), Y338 (1), Y340 (5), Y344 (6), Y346 (1), Y348 (2), Y349 (15), Y405 (1), Y579 (3), Y838 (1), Y839 (4), Y846 (1)

* Ill-defined deaths include ICD-10 codes R00-R99 (*Symptoms, signs and abnormal clinical and laboratory findings, not elsewhere classified*) plus ICD-10 codes E86 (*Volume depletion*), E870 (*Hyperosmolality and hypernatraemia*), E872 (*Acidosis*), E873 (*Alkalosis*) and E874 (*Mixed disorder of acid-base balance*).

Table S3. Sensitivity 'two-sample' MR analyses for all-cause mortality at ages 35-74 years, using SNP-to-BMI associations derived from a trans-ancestry GWAS of China Kadoorie Biobank and UK Biobank

	Death RR (95% CI) per 5 kg/m² higher genetically-predicted BMI	
	**Men**	**Women**
Inverse-variance weighted (IVW)[a]	1.55 (1.37, 1.76)	1.53 (1.40, 1.67)
MR-Egger[b]	1.53 (1.06, 2.22)	1.76 (1.36, 2.29)
Weighted median[c]	1.68 (1.35, 2.08)	1.52 (1.32, 1.77)
Outlier corrected IVW (Modified Q statistic)[d]	1.55 (1.37, 1.76)	1.53 (1.40, 1.67)

RR (95% CI) shown per 5 kg/m² higher genetically-predicted BMI using various 'two sample' MR approaches (all adjusted for age, age² and the first 7 genetic principal components). For each approach the SNP-BMI effect estimates and their standard errors are taken from trans-ancestry meta-analysis of China Kadoorie Biobank with UK Biobank[w6], while the SNP-mortality effect estimates and their standard errors are taken from MCPS. For all approaches, the 724 SNPs are aligned so that the SNP-BMI associations are strictly positive. Details of the four methods can be found in https://cran.r-project.org/web/packages/MendelianRandomization/MendelianRandomization.pdf. Brief details underlying assumptions are provided below.

[a] An inverse variance weighted (IVW) linear regression of the 724 SNP-mortality effects on the 724 (aligned) SNP-BMI effects is performed in which the regression line is forced through the origin. It relies on the assumption of no 'horizontal pleiotropy' (ie, it assumed that all of the SNPs affect mortality only through BMI and not through any other mechanism).

[b]A modification of the IVW approach in which the regression is not forced through the origin. The intercept provides an estimate of net horizontal pleiotropic effects under the assumption that any such effects are uncorrelated with the SNP-exposure effects (known as the InSIDE assumption). For each RR shown in the above table, the intercept term was non-significant at the 5% level.

[c]The weighted median approach applies the ratio method to each SNP, orders the results, assigns them normalised inverse-variance weights (to capture the 'information content' of each SNP), then selects the ratio estimate at which the cumulative information content first passes half of the total information content. It relies on the assumption that at least half of the statistical information on the SNP-BMI associations comes from valid instrumental variables. It is more robust to individual genetic variants with strongly outlying causal estimates compared with the IVW and MR-Egger methods.

[d]Outlier-corrected IVW (MR-PRESSO) applies the IVW approach but to a reduced set of SNPs in which any SNPs contributing significant heterogeneity (after Bonferroni-correction) to the estimated IVW slope are removed, and then the IVW approach is re-applied to the remaining SNPs.

Supplementary methods

Study design and participants

Details of the MCPS design, methods and participants have been described previously.[w1] In brief, between 1998 and 2004 households in two urban districts of Mexico City (Coyoacán and Iztapalapa) were visited and residents aged 35 or older were invited to participate in the study. Of the 112,333 households with eligible residents, at least one individual from 106,059 (94%) households participated. Of the 159,755 participants recruited, about two thirds were women and one third men, reflecting the fact that the majority of home visits were carried out during working hours when women were more likely to be at home. Ethics approval for the study was obtained from the Mexican Ministry of Health, the Mexican National Council for Science and Technology, and the University of Oxford, UK. All participants provided written informed consent.

Data collection

During household visits trained nurses administered electronic questionnaires recording sociodemographic and lifestyle factors, current medications, and medical history. Calibrated electronic scales, stadiometers, and nonstretchable tape were used to measure weight (to the nearest 0.1 kg), height (to the nearest 0.1 cm), and waist and hip circumference (also to the nearest 0.1cm), respectively. Body mass index was calculated as weight in kilograms divided by the square of height in metres. Blood pressure was measured with the participant seated. A 10-mL non-fasting venous blood sample was collected into an EDTA vacutainer, separated into plasma and buffy coat aliquots and stored long-term at -150°C. Glycosylated haemoglobin (HbA1c) was measured in buffy coat samples using validated high-performance liquid chromatography[w2] on HA-8180 analysers with calibrators traceable to International Federation of Clinical Chemistry standards.[w3] Previously-diagnosed diabetes was defined as a self-reported doctor diagnosis or regular use of a diabetes medication, while undiagnosed diabetes was defined as a baseline HbA1c concentration ≥6.5% (≥48 mmol/mol) in someone without previously-diagnosed diabetes. Genotyping was performed in all participants with version 2 of the Illumina Global Screening Array.[w4] Genetic imputation, and quality control procedures including individual-level missingness, departures from Hardy-Weinberg equilibrium, and Mendel errors were conducted as previously described.[w5] Per-individual proportions of Indigenous, European, African and East Asian inherited ancestry (i.e., the different proportions of their genomes descended from previously discrete subpopulations), were estimated.[w5]

Exclusion criteria

All analyses excluded those with missing genetic data (or genetic data that did not pass quality control)[w5], uncertain mortality linkage (defined as a ≥1 year one discrepancy in a participant's date of birth as recorded at the baseline survey compared with the matched death certificate) or recorded height or weight that was considered likely to reflect a data entry error (weight <30 or >200 kgs, height <120 cms).

Instrumental variable for BMI

The main analyses used a genetic instrument score for BMI (BMI-GS) using results from a trans-ancestry meta-analysis of results from genome-wide association studies of a large East Asian and a large European population.[w6] Specifically, 907 conditionally-independent genetic variants associated with BMI at genome-wide significance level ($P \leq 5 \times 10^{-8}$) and LD-clumped at an r^2 threshold of 0.05 were initially selected from the trans-ancestry meta-analysis. Of these, 123 variants were unavailable in the MCPS genetic imputed data (as direct matches or proxies based on a linkage disequilibrium [LD] at an $r^2 \geq 0.8$) and three variants were ambiguous as the allelic strand could not be harmonized. For the remaining 781 variants (listed in **Supplementary Data Item 1**) further clumping of variants in linkage-disequilibrium (based on $r^2 \geq 0.001$ and a clumping window of ±5 Mb) left 724 variants remaining for inclusion in the trans-ancestry polygenic instrument. In sensitivity analyses, an alternative BMI-GS was constructed from European ancestry populations published by the GIANT Consortium.[w7] This instrument included 772 conditionally independent genome-wide significant genetic variants LD-clumped at an r^2 threshold of 0.05, of which six could not be directly or approximately matched with variants in MCPS. For the remaining 766 variants (listed in **Supplementary Data Item 2**) further clumping at an r^2 threshold of 0.001 left 612 SNPs remaining

for inclusion in the GIANT polygenic instrument. Of these 612 variants, 145 overlapped (directly or through LD $r^2 \geq 0.8$) with the 724 variants included in the trans-ancestry BMI-GS. Each participant's BMI genetic score was calculated as the linear combination of the SNP-dosages multiplied by their weights (which were the same irrespective of sex for the trans-ancestry BMI-GS but sex-specific for the GIANT BMI-GS).

Instrumental variable for diabetes
For the purpose of estimating the extent to which the causal effect of BMI on mortality was mediated by diabetes, a genetic score for diabetes was derived from the Type 2 Diabetes Global Genomics Initiative trans-ancestry meta-analysis of genome-wide association studies.[w8] Of the 1289 genome-wide significant and independent (clumped at $r^2 \geq 0.05$) variants, we excluded 234 variants that were unavailable in MCPS (as direct matches or proxies based on an LD $r^2 \geq 0.8$), a further 242 after clumping at an r^2 threshold of 0.001, and 58 that were in high LD ($r^2 \geq 0.8$) with variants from the trans-ancestry BMI-GS (**Supplementary Data Item 3**), leaving 755 variants for the diabetes GS.

Main regression models and covariate adjustment
The Wald ratio method[w9] was used to assess the causal relevance of BMI to cause-specific mortality. For this ratio, Cox regression was used to estimate the association between the trans-ancestry BMI-GS and mortality, with adjustment for age-at-risk (in 5-year-bands) and the first seven genetic principal components (PCs). Adjustment for age-at-risk rather than baseline age was done to provide a more complete adjustment for the potential confounding effects of age (as well as for consistency with previous epidemiological analyses of BMI and mortality).[w10] For the denominator of the Wald ratio, linear regression estimated the association between the trans-ancestry BMI-GS and baseline BMI, with adjustment for age, age-squared and the first seven genetic PCs. Seven PCs were chosen (rather than, for example, ten) because higher PCs in the MCPS population display localized genomic regions with increased loadings due to long-range linkage-disequilibrium.[w5] In sensitivity analyses we performed a range of additional 'two-sample' MR approaches (inverse variance weighted, MR-Egger, weighted median and outlier corrected inverse variance weighted [MR-PRESSO]).[w11] These analyses used the SUGEN software[w12] (which accounts for relatedness) to estimate the mortality log hazard ratio associated with variant. A description of these 2-sample MR approaches and their assumptions is provided in **Table S3**.

Mortality follow-up
Death registration in Mexico City is reliable and complete, with almost all deaths certified medically.[w13] Participants are followed for cause-specific mortality through probabilistic linkage (based on name, including phonetic coding of names, age, and sex) to the Mexican System for Epidemiologic Death Statistics (Subsistema Epidemiológico y Estadístico de Defunciones or SEED) electronic death registry in Mexico City, administered by the Ministry of Health. Field-worker follow-up of more than 7000 deaths matched in this way confirmed that the match was correct in more than 95% of cases. Diseases recorded on death certificates are coded using the International Statistical Classification of Diseases and Related Health Problems, Tenth Revision, with subsequent review by study clinicians to recode, when necessary, the underlying cause of death.[w14]

Participant deaths were tracked from recruitment (1998 to 2004) until October 1, 2022. In total, results are shown for **52 mortality outcomes**: the 38 individual specific causes shown in **Figure 3** (with RRs represented by squares and 95% CIs shown through them); and the 14 composite outcomes shown in **Figure 2** and/or **Figure 3** (cardiac, cerebrovascular, other vascular, any vascular, renal, hepatobiliary, any metabolic, any vascular-metabolic, cancer, respiratory, infective, external/other medical/ill-defined, any non vascular-metabolic, and all-cause). Most analyses focused on death before age 75 years (considered to be 'premature') but additional analyses of deaths at older ages (75 to 89 years) are also provided.

Appendix references

w1. Tapia-Conyer R, Kuri-Morales P, Alegre-Díaz J, et al. Cohort profile: the Mexico City Prospective Study. Int J Epidemiol 2006; 35(2): 243-9

w2. Youngman LD, Clark S, Manley S, Peto R, Collins R. Reliable measurement of glycated hemoglobin in frozen blood samples: implications for epidemiologic studies. Clin Chem 2002; 48:1627-9.

w3. International Federation of Clinical Chemistry. Standardization of HbA1c. NGSP (online http://www.ngsp.org/docs/IFCCstd.pdf).

w4. Illumina Infinitum Global Screening Array (https://www.illumina.com/content /dam/illumina-marketing/documents/products/datasheets/infinium-global-screening-array-data-sheet-370-2016-016.pdf)

w5. Ziyatdinov A, Torres J, Alegre-Díaz J, et al. Genotyping, sequencing and analysis of 140,000 adults from Mexico City. Nature 2023; 622: 784-93

w6. Fairhurst-Hunter Z, Lin K, Millwood IY, Pozarickij A, Chen T-T, Torres JM, et al. Trans-ancestry meta-analysis improves performance of genetic scores for multiple adiposity-related traits in East Asian populations. MedRxiv. 2022:2022.07.05.22277254.

w7. Pulit SL, Stoneman C, Morris AP, Wood AR, Glastonbury CA, Tyrrell J, et al. Meta-analysis of genome-wide association studies for body fat distribution in 694 649 individuals of European ancestry. Human Molec Gen. 2019; 28: 166-74.

w8. Suzuki K, Hatzikotoulas K, Southam L, et al. Genetic drivers of heterogeneity in type 2 diabetes pathophysiology. Nature 2024; 627: 347-57.

w9. Burgess S, Thompson SG: Mendelian Randomization: Methods for using Genetic Variants in Causal Estimation. Taylor & Francis Group; 2015

w10. Gnatiuc L, Alegre-Diaz J, Wade R, Ramirez-Reyes R, Tapia-Conyer R, Garcilazo-Avila A, et al. General and Abdominal Adiposity and Mortality in Mexico City: A Prospective Study of 150 000 Adults. Ann Int Med. 2019;171:397-405

w11. Burgess S, Bowden J, Fall T, Ingelsson E, Thompson SG. Sensitivity Analyses for Robust Causal Inference from Mendelian Randomization Analyses with Multiple Genetic Variants. Epidemiology (Cambridge, Mass). 2017;28:30-42

w12. Lin D-Y, Tao R, Kalsbeek WD, Zeng D, Gonzalez F, Fernández-Rhodes L, et al. Genetic association analysis under complex survey sampling: the Hispanic Community Health Study/Study of Latinos. Am J Hum Gen. 2014; 95: 675-88

w13. Mikkelsen L, Phillips DE, AbouZahr C, et al. A global assessment of civil registration and vital statistics systems: monitoring data quality and progress. Lancet 2015; 386: 1395-406.

w14. Alegre-Diaz J, Herrington W, Lopez-Cervantes M, et al. Diabetes and Cause-Specific Mortality in Mexico City. NEJM 2016; 375: 1961-71.
